# Exploring the correlation of metabolites changes and microbial succession in solid-state fermentation of Sichuan Sun-dried vinegar

**DOI:** 10.1186/s12866-023-02947-1

**Published:** 2023-07-24

**Authors:** Ke Dong, Weizhou Li, Qiuhong Xu, Zehui Hong, Shirong Zhang, Baochao Zhang, Yating Wu, Haojiang Zuo, Jiazhen Liu, Ziwen Yan, Xiaofang Pei

**Affiliations:** 1 West China School of Public Health and West China Fourth Hospital, Sichuan University, 16#, Section 3, Renmin Nan Road, Chengdu, 610041 PR China; 2 Food Safety Monitoring and Risk Assessment Key Laboratory of Sichuan Province, Department of Public Health Laboratory Sciences, West China School of Public Health, Sichuan University, Chengdu, 610041 PR China; 3 West China-PUMC C. C. Chen Institute of Health, Sichuan University, Chengdu, 610041 PR China; 4 Zigong Qiantian Baiwei Food Co., Ltd, Zigong, 643200 PR China

**Keywords:** Sichuan Sun-dried vinegar, Microbial community, Fermentation, Metabolites, Correlation analysis

## Abstract

**Background:**

The traditional Sichuan Sun-dried vinegar (SSV) with unique flavor and taste is believed to be generated by the solid-state fermentation craft. However, how microorganisms and their metabolites change along with fermentation has not yet been explored.

**Results:**

In this study, our results demonstrated that the middle and late stages of SSV fermentation were the periods showing the largest accumulation of organic acids and amino acids. Furthermore, in the bacterial community, the highest average relative abundance was *Lactobacillus* (ranging from 37.55 to 92.50%) in all fermentation stages, while *Acetobacters* ranked second position (ranging from 20.15 to 0.55%). The number of culturable lactic acid bacteria is also increased during fermentation process (ranging from 3.93 to 8.31 CFU/g). In fungal community, *Alternaria* (29.42%), *Issatchenkia* (37.56%) and *Zygosaccharomyces* (69.24%) were most abundant in different fermentation stages, respectively. Interestingly, *Zygosaccharomyces*, *Schwanniomyces* and *Issatchenkia* were first noticed as the dominant yeast genera in vinegar fermentation process. Additionally, spearman correlation coefficients exhibited that *Lactobacillus*, *Zygosaccharomyces* and *Schwanniomyces* were significant correlation with most metabolites during the fermentation, implying that these microorganisms might make a significant contribution to the flavor formation of SSV.

**Conclusion:**

The unique flavor of SSV is mainly produced by the core microorganisms (*Lactobacillus*, *Zygosaccharomyces* and *Schwanniomyces*) during fermentation. This study will provide detailed information related to the structure of microorganism and correlation between changes in metabolites and microbial succession in SSV. And it will be very helpful for proposing a potential approach to monitor the traditional fermentation process.

**Supplementary Information:**

The online version contains supplementary material available at 10.1186/s12866-023-02947-1.

## Introduction

**Fig. 1:**
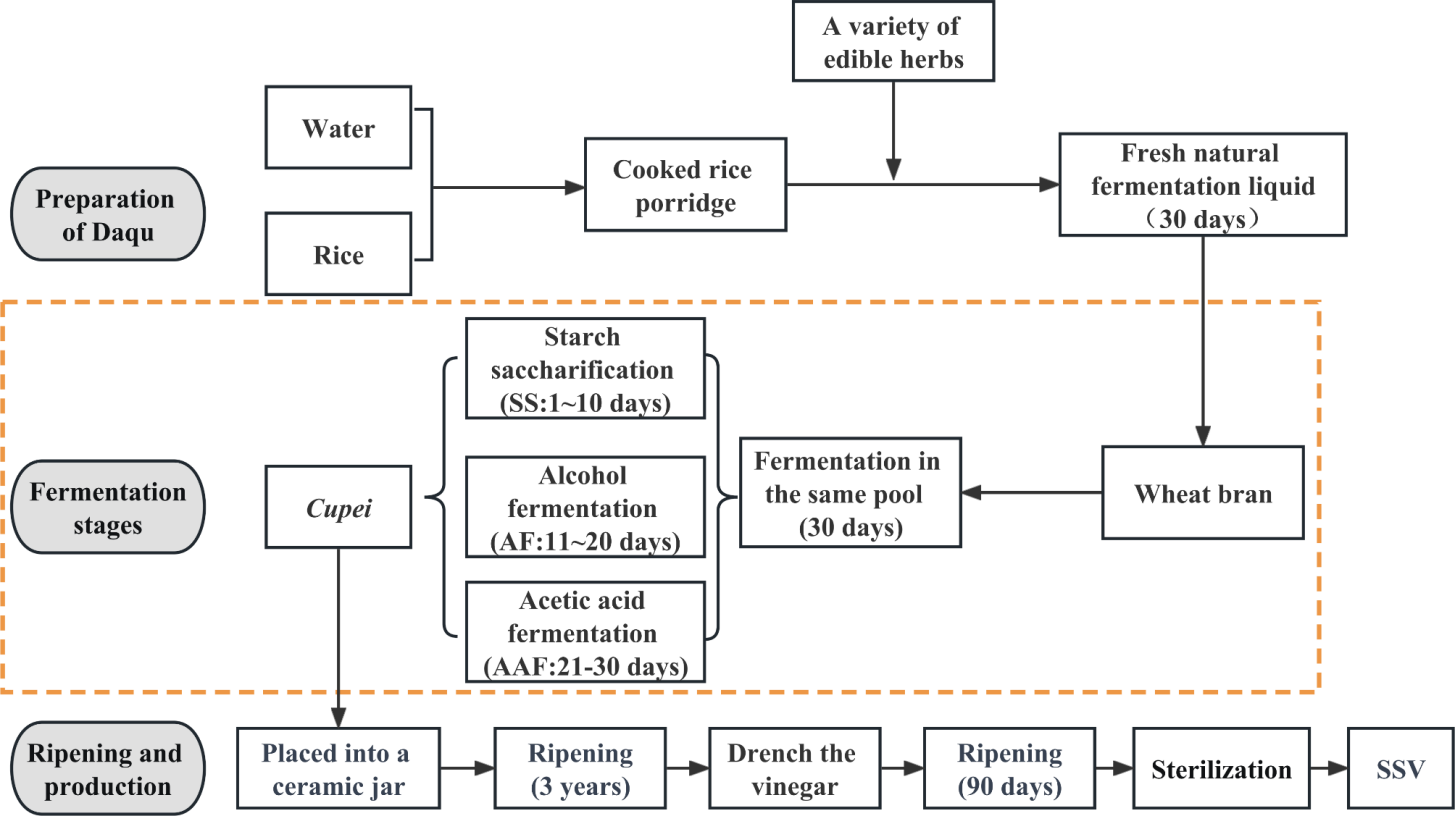
Process flow chart of SSV production

Vinegar is a worldwide traditional fermented condiment and a crucial material in the food industry [1, 2]. It has been produced and consumed as a commercial product with a history of approximately 5000 years [3]. Sichuan Sun-dried vinegar (SSV), produced in Sichuan Province, is quite famous because of unique flavor and soft taste with rich nutrition, which are believed to be generated by the special solid-state fermentation (SSF)craft. Reportedly, SSF is a fermentation process that exhibits characteristics similar to spontaneous fermentation, involving a long-term aging process and yielding multiple metabolites [4, 5]. It can enhance the nutritional value and bioactivity of the raw materials utilized in fermentation, as well as endow the resulting vinegar with a distinct and mellow flavor profile. Notably, the major flavor metabolites produced through this process, alongside organic acids and amino acids, exhibit various health benefits, including antioxidant, antihypertensive, anti-obesity, antidiabetic, and antimicrobial activities [6, 7]. These attributes have led to consumer approval of SSF vinegar as a healthful and flavorful dietary option. Compared with other Chinese vinegars, the fermentation craft of SSV (Fig. 1) is different in three aspects. Firstly, wheat bran is used as the only major ingredient during the fermentation. Secondly, the fermentation starter (*Daqu*) is not powder, but fresh natural fermentation liquid made from cooked rice porridge supplemented with a variety of edible herbs such as patchouli, Chinese yam, etc. Previous studies have reported that *Daqu* used in vinegar fermentation contained a rich variety of microorganisms, primarily including molds/yeasts and lactic acid bacteria (LAB) [2, 8]. Thirdly, the entire fermentation process, including starch saccharification (SS), alcohol fermentation (AF) and acetic acid fermentation (AAF), was conducted simultaneously in the same pool for 30 days, and the *Cupei* was manually stirred every morning (by facilitating the exchange between the upper and lower layers of *Cupei*) to ensure adequate oxygen supply, promote heat release, enhance the yield of acetic acid and maintain gas exchange balanced [5, 9]. But how the microbial community changes of this fermentation process has never been explored.

Previous studies demonstrated that different fermentation craft of Chinese traditional vinegars may lead to variation in microbial community composition [1, 8, 10–12]. For example, during shanxi aged vinegar fermentation process, the SS and AF stages were carried out simultaneously after mixing with *Daqu* powder and water, and then the last batch of *Cupei* was added to start the AAF stage. Nie et al. also found that LAB increased in the AF stage, then decreased in AAF stage, and the abundance of acetic acid bacteria (AAB) (50.9%) was higher than that of LAB [5]. Whereas, during the Tianjing duliu vinegar fermentation process, *Daqu* usually mixed with raw materials for SS, then adding water and auxiliary materials go on AF and AAF stages, respectively. The results showed AAB increased continuously in the early fermentation stage, but the abundance of LAB (> 80%) was higher than that of AAB in the late fermentation stage [8]. Furthermore, the taste and flavor of vinegar were determined by the abundant metabolites such as free amino acids (FAAs), organic acids (OAs), volatile compounds and other bioactive constituents, which were generated by the microorganisms [2]. Therefore, the correlation between metabolites changes and microbial succession in traditional Chinese vinegars were also studied. Nie et al. found that *Saccharomyces*, *Komagataeibacter* and *Acinetobacter* were positively correlated with alcohol or acetic acid production in Shanxi aged vinegar by using 454 high-throughput sequencing, GC-MS and HPLC [5], and they also found that LAB had important influences on the flavor and taste of vinegars [8]. Mei et al. exhibited that *Lactobacillus*, *Acetobacter* and *Candida* were closely related with volatiles and organic acids in Sichuan bran vinegar by using Illumina-MiSeq sequencing, HPLC and HS-SPME-GC-MS as well [13]. Since the craft of SSV is quite different from other vinegars, exploring the correlation of metabolites changes and microbial succession in the fermentation process will be very helpful to understand the essence mechanism of this kind of solid-state fermentation. To the best of our knowledge, no research has been reported on the correlation between changes in metabolites and microbial succession, as well as the composition of microbial communities during different fermentation stages of SSV.

Therefore, in this study, Illumina high-throughput sequencing technology and cultivation method were applied to investigate the structure of microbial communities and the numeration of culturable microorganisms in the three fermentation stages, respectively. Moreover, HPLC and chemical methods were performed to determine metabolites and flavor components, and the potential correlations between dominant microbiota and major metabolites of SSV were explored. This study will provide detailed information related to the structure of microorganism and correlation between changes in metabolites and microbial succession. And it will be very helpful for proposing a potential approach to monitor the traditional fermentation process. Furthermore, since recent evidence suggested that some kind of traditional vinegar have several types of therapeutic effects like antioxidant, hypotensive, hypoglycemic and cholesterol-lowering activities [6, 7]. Thus, our study will also be helpful to further investigate the medicinal benefits of SSV.

## Results and discussion

### Changes in Physiochemical features during fermentation process

**Fig. 2:**
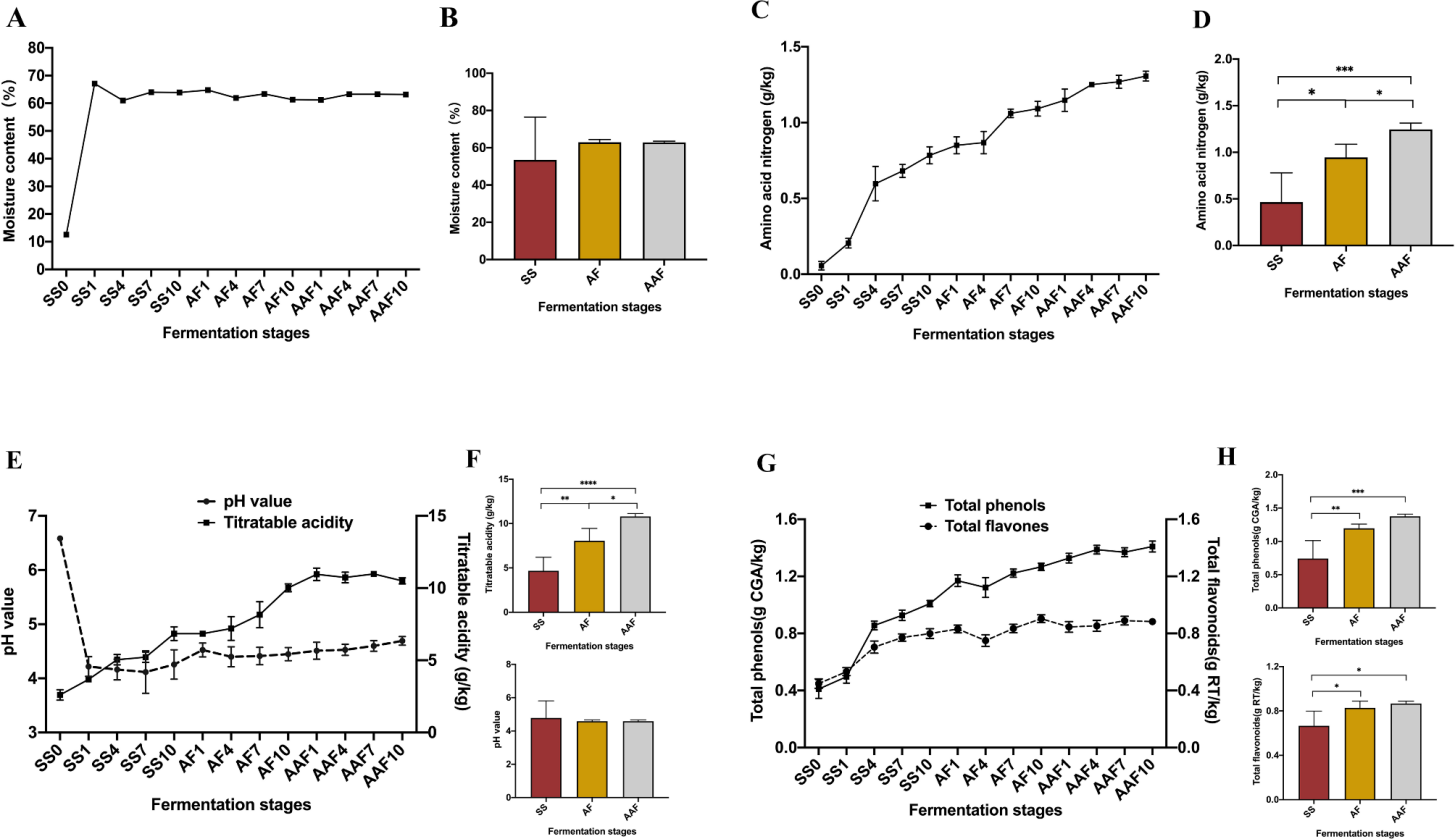
Physicochemical characteristics of *Cupei* samples in different fermentation stages. Changes in the moisture (**A, B)**, amino nitrogen (**C, D**), pH value and titratable acidity (**E, F**), total phenols and total flavonoids and pigment (**G, H**). Data were given as mean ± standard deviations. *P < 0.05; **P < 0.01; ***P < 0.001

The changes of physicochemical features, including moisture, amino acid nitrogen, titratable acidity and pH of *Cupei* samples were illustrated in Fig. 2 and Table S1. The moisture content of *Cupei* was maintained at 60%~65% during the fermentation process (Fig. 2A and B), which was due to the better water absorption and holding capacity of the wheat bran, ensured the high moisture content of *Cupei*. The amino acid nitrogen, titratable acidity gradually increased from 0.06 g/kg to 1.31 g/kg, and from 2.68 g/kg to 10.50 g/kg, respectively and the contents were statistically significant in three fermentation stages (Fig. 2C ~ F). This could be attributed to the complex interaction and alternation of multiple microorganisms, leading to the generation of metabolites and the degradation of nitrogen-containing compounds [14]. Conversely, the pH values decreased sharply from the 0d to 1d (6.58 ~ 4.22) after the addition of *Daqu* and were maintained between 4.22 and 4.69 during fermentation stages (Fig. 2E). Compared with other cereal vinegars, the pH values were maintained between 3.0 and 3.8 during fermentation stages [8, 15, 16]. The fluctuation of pH value and titratable acidity may probably due to the production or consumption of organic acids (lactic acid and acetic acid) by multiple bacterial and fungal genera during the fermentation process [5, 17]. These physiochemical factors were not only the indicators of normal fermentation but also the potential vital environmental drivers in the assembly of microbial community of fermented foods [18, 19].

### Changes of Organic acids during fermentation process

**Fig. 3:**
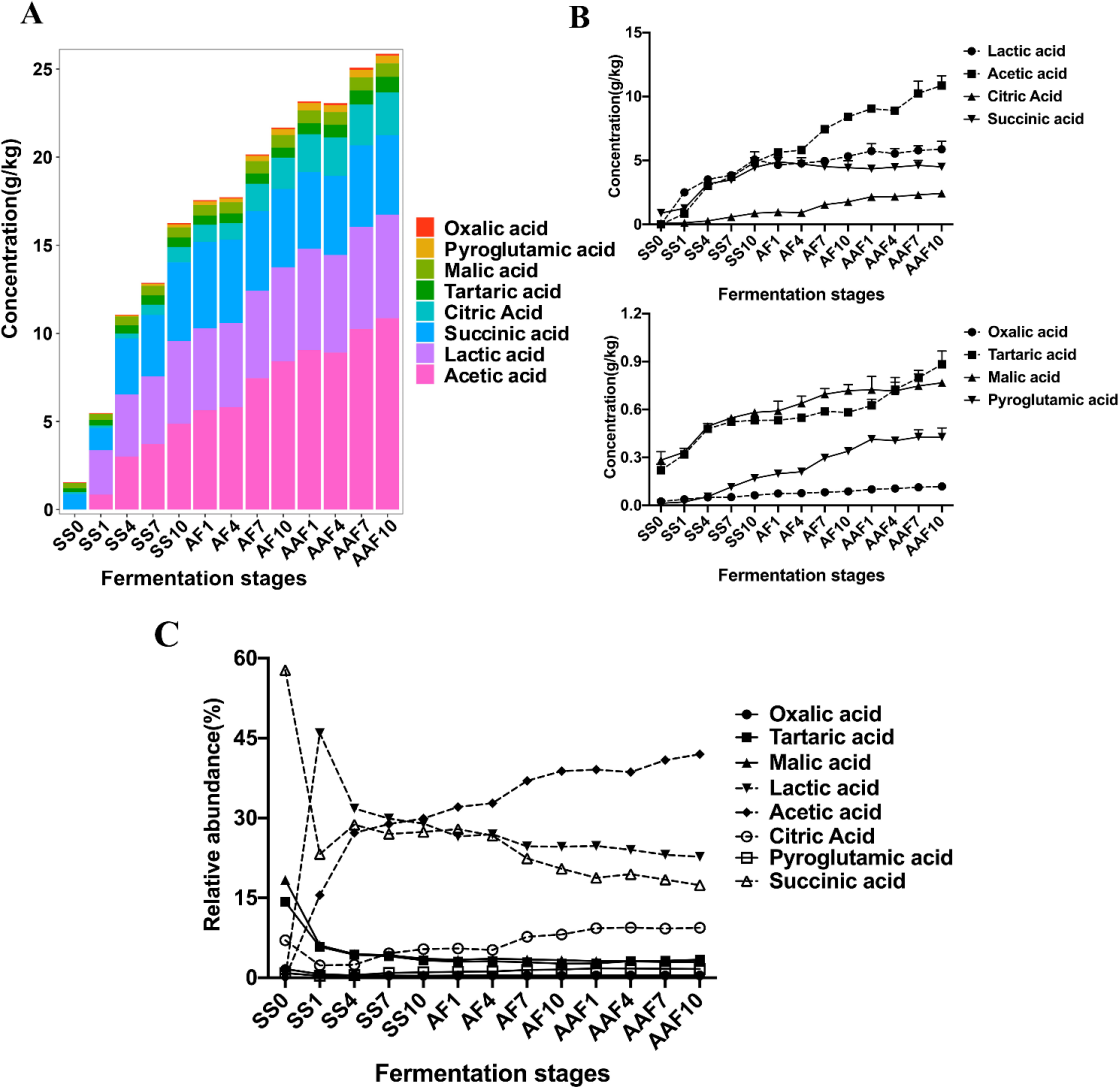
Changes of OAs in *Cupei* samples. Changes of total organic acid content (**A**), eight organic acids content (**B**), and the proportion of eight organic acids (**C**) in different fermentation stages. Data were given as mean ± standard deviations

Organic acids (OAs) are one of the main flavor substances of vinegar, which are converted from proteins, starches and fats in the raw materials through the action of microorganisms during fermentation [20]. The types and contents of OAs are strongly related to the taste and quality of vinegar. In this study, 8 OAs in the fermentation process of SSV were quantified with calibration curves and the variations were presented in Fig. 3 and Table S2. During the whole fermentation process, the total level of OAs showed a rising trend, from 1.54 ± 0.30 g/kg at the 0d (SS0) to 24.29 ± 1.21 g/kg at the 30d (AAF10) (Fig. 3A). Among these OAs, acetic acid, lactic acid and succinic acid were found to be the top 3 OAs in terms of their content, accounting for more than 80% of the total OAs. The acetic acid concentration increased from 0.85 ± 0.05 g/kg at the 1d (SS1) to 10.86 ± 0.62 g/kg at the end of fermentation (AAF10). The content of lactic acid was higher than that of acetic acid in SS fermentation stage, and showed an increasing trend in the first 21d of fermentation, and then decreased slightly, remained stable in the AAF stage. Acetic acid and lactic acid were the predominant OAs in cereal vinegar, and lactic acid was benefit to balance the sourness stimulate taste of acetic acid [21]. Similarly, the change trend of succinic acid was basically consistent with that of lactic acid (Fig. 3B). These findings were in line with Shanxi aged vinegar and Tianjin duliu mature vinegar [5, 8]. Besides, other OAs with low content in SSV were also increased during the fermentation process, such as oxalic acid, malic acid, citric acid, tartaric acid and pyroglutamic acid. Among them, oxalic acid, malic acid, and pyroglutamic acid showed a slow upward trend. However, the proportion of these 5 OAs decreased from 42.24% at the beginning of fermentation to 17.88% at the end of fermentation (Fig. 3C). On the other hand, these OAs were intermediate metabolites of the tricarboxylic acid cycle, which were used as substrates and enter other metabolic pathways, and thus could not accumulate in large quantities during the fermentation process [22]. However, the content of citric acid increased significantly during the fermentation process, which might be attributed to the metabolites of AAB and LAB [4, 5]. In addition, these bacteria could also utilize other organic acids to produce citric acid through the tricarboxylic acid cycle pathway [22]. The presence of these OAs reduced the influence of acetic acid and increased the softness of vinegars [23].

### Changes of free amino acids during fermentation process

**Fig. 4:**
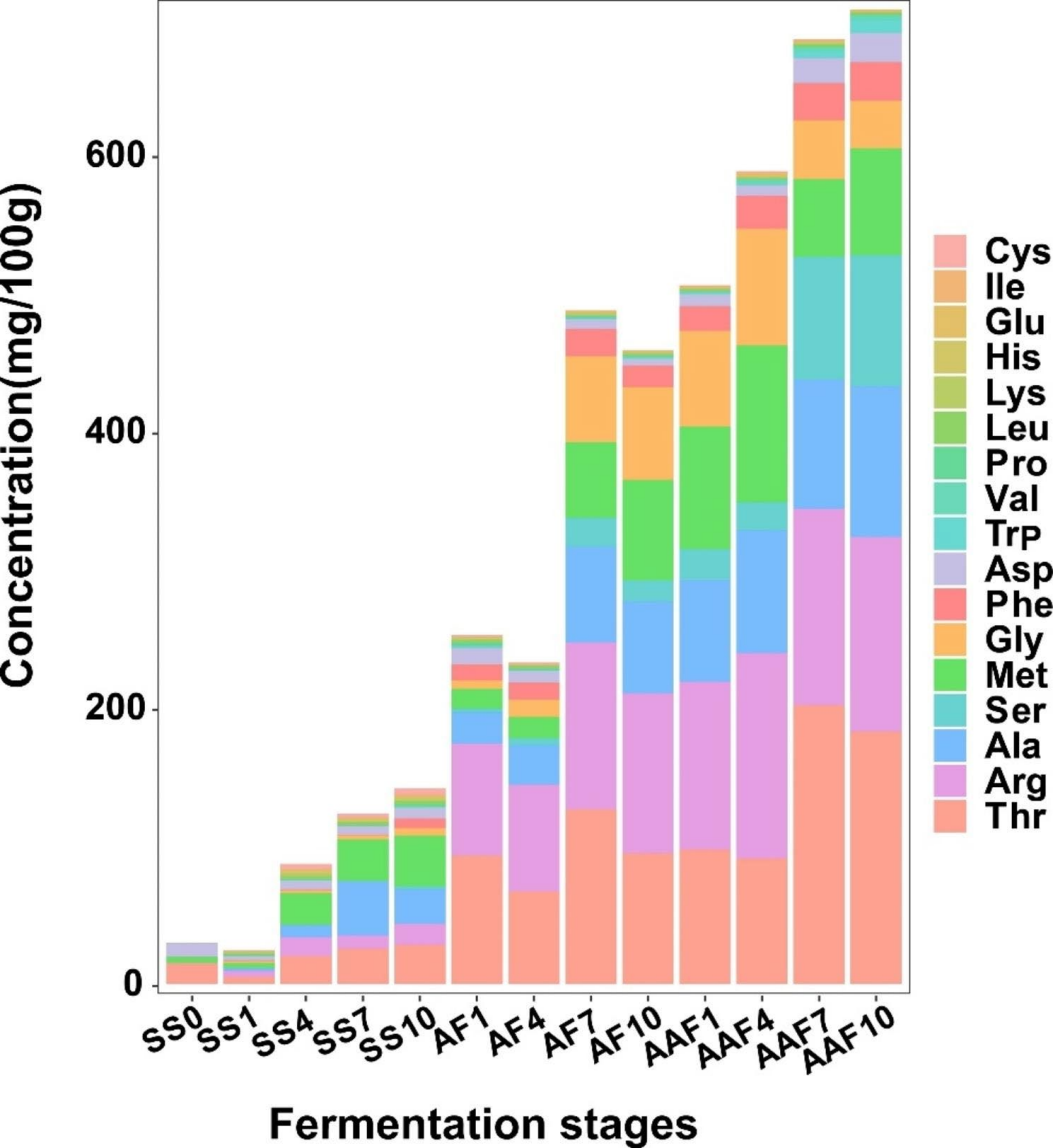
Changes of FAAs in *Cupei* samples

Free amino acids (FAAs) are important flavor and nutrient components of vinegars, as well as serve as precursors for the formation of other flavor substances [23]. As described in Fig. 4 and Table S3, the contents of total FAAs accumulated during the whole fermentation process, from 29.64 ± 4.19 mg/100 g at the 0d (SS0) to 705.35 ± 56.03 mg/100 g at the 30d (AAF10). The contents of FAAs were relatively more abundant at the middle and late stages of fermentation. In the raw material sample (SS0), only three kinds of free amino acids were detected, which were methionine (Met), threonine (Thr) and aspartic acid (Asp). After 7 days of fermentation, 17 FAAs were detected in all *Cupei* samples. Among them, Thr, arginine (Arg) and alanine (Ala) were the most abundant. The contents of these three amino acids increased significantly in the middle and late stages of fermentation, accounting for more than 50% of the total FAAs, and reached 61.35% at the end of fermentation. In contrast with *Monascus* rice vinegar, 3 FAAs with higher content were glutamic acid (Glu), Ala and leucine (Leu) during the fermentation process, whose total contents accounted for more than 43% of the total FAAs. In addition, the total content of serine (Ser), Met, glycine (Gly), phenylalanine (Phe), Asp and tryptophan (Trp) at the end of fermentation accounted for 37.61% of the total FAAs. The remaining eight amino acids, Glu, cystine (Cys), lysine (Lys), proline (Pro), Leu, valine (Val), Isoleucine (Ile) and histidine (His), showed dynamic changes during fermentation, and their contents were maintained at a low level. The variations of FAAs in SSV were similar to *Monascus* rice vinegar, where FAAs increased rapidly on middle and late fermentation stages [6]. The accumulation of FAAs not only improved the flavor of vinegars, but also provided the precursors necessary for the Maillard reaction in the subsequent aging stage, which promoted the formation of flavor and functional substances in Chinese traditional vinegars [23].

Furthermore, the taste of SSV also be influenced by the FAAs. Generally, FAAs are classified into umami, sweetness and bitterness flavors [24, 25]. As described in Table 1, the content of total sweet-taste FAAs was the highest, stabilizing at around 50% of total amino acids during the entire fermentation process, followed by bitter-taste FAAs, while the umami-taste FAAs were the lowest, accounting for only about 3% of the total FAA. And besides umami-taste amino acids, the content of sweet-taste and bitter-taste amino acids in the three stages of fermentation were statistically significant. In contrast with *Monascus* rice vinegar, the bitter-taste amino acids were the most abundant, accounting for about 50% [6]. The reason for this difference might be the different fermentation craft of the two vinegars [1, 2, 8]. *Monascus* vinegar is fermented by liquid-state fermentation craft, while SSV is fermented by solid-state fermentation craft.

**Table 1:** Concentrations of FAAs in different flavor among *Cupei* samples

Samples	FAAs* (mg/100 g)		
	Umami	Sweet	Bitter
SS0	9.93 ± 4.68	15.20 ± 5.32	4.51 ± 1.58
SS1	3.31 ± 1.3	10.55 ± 1.71	10.78 ± 1.57
SS4	7.64 ± 2.69	36.05 ± 6.79	43.24 ± 8.10
SS7	7.00 ± 2.75	71.79 ± 14.49	44.52 ± 10.07
SS10	8.8 ± 3.35	66.87 ± 11.01	65.92 ± 12.03
AF1	12.37 ± 5.5	128.65 ± 31.3	111.49 ± 27.03
AF4	8.94 ± 3.87	115.51 ± 22.58	108.32 ± 25.93
AF7	8.56 ± 2.87	280.69 ± 44.57	198.34 ± 41.91
AF10	6.28 ± 1.90	245.81 ± 36.86	206.74 ± 42.79
AAF1	9.51 ± 3.88	264.35 ± 38.28	231.96 ± 46.63
AAF4	9.05 ± 3.12	286.13 ± 41.34	293.29 ± 57.69
AAF7	19.54 ± 8.12	433.53 ± 68	230.91 ± 48.52
AAF10	22.38 ± 9.55	431.65 ± 64.29	251.32 ± 50.17
Mean^SS^	7.34 ± 2.25	40.09 ± 25.42^a^	33.79 ± 22.91^a^
Mean^AF^	9.04 ± 2.18	192.67 ± 71.80^b^	156.22 ± 46.43^b^
Mean^AAF^	15.12 ± 5.93	353.92 ± 79.05^c^	251.87 ± 25.26^c^

*The results are expressed as mean ± standard deviation with three triplicates for each sample. Umami FAAs include Asp, Glu and Lys; Sweet FAAs include Ala, Gly, Cys, Pro, Trp, Ser and Thr; Bitter FAAs include Phe, Met, Arg, Leu, Val, Ile and His. ^SS^ SS fermentation stage samples; ^AF^ AF fermentation stage samples; ^AAF^ AAF fermentation stage samples; ^a^ SS fermentation stage samples vs. AF fermentation stage samples (*P* < 0.05); ^b^ AF fermentation stage samples vs. AAF fermentation stage samples (*P* < 0.05); ^c^ AAF fermentation stage samples vs. SS fermentation stage samples (*P* < 0.05)

### Changes of total phenols and total flavonoids during fermentation process

Since phenols are not only important flavor substances in Chinese traditional cereal vinegars [14], but also are bioactive components with antioxidant activity [26, 27]. In this study, we determined the level of total phenols (TP) and total flavonoids (TF), and the results were presented in Fig. 2. The contents of TP and TF gradually increased during the fermentation process (Fig. 2G and H). In the raw material sample (SS0), the content of TP was 0.47 g CAE/kg, then increased, reaching 1.41 g CAE/kg at the end of fermentation. Apart from the source of raw materials, the formation of phenols was also associated with the fermentation process. Previous studies showed that fermentation improved the content of phenols, and the aroma of vinegars was also correlated with the phenolic substances [28, 29]. Therefore, Gao et al. further determined the phenolic components in the fermentation process of *Monascus* rice vinegar, which exhibited that ferulic acid content was the highest, accounting for about 50% of the TPs during the fermentation process, followed by vanillic acid (ranging from 15 to 30%) [6]. Besides, the variation trend of TF content was similar to that of TP content during fermentation. On the 20th day, the content of total flavonoids reached the highest (0.90 g RTE/kg), and then remained stable from 21d to 30d (0.85 ~ 0.89 g RTE/kg). These results were in accordance with the accumulation of TP and TF during fermentation of Zhenjiang vinegar and *Monascus* rice vinegar [6, 15].

### Alpha diversity analysis during fermentation process

To obtain an overview of community diversity and succession of SSV fermentation, 13 *Cupei* samples from different fermentation stages were analyzed using Illumina high-throughput sequencing technology. The coverage rate of all *Cupei* samples was greater than 99.9%, indicating that the identification of the microorganisms (bacterial and fungi) was highly possible. And the sequencing depth of the samples was sufficient according to the rarefaction and rank - abundance curves (Fig. S1), which could meet the requirement for subsequent bioinformatic analysis. The number of bacteria ASVs/OTUs (Operational Taxonomic Units) of all samples were 986, while fungal ASVs/OTUs were 432. And a total of 142 bacterial genera and 132 fungal genera were identified in SSV *Cupei* samples. Compared with Shanxi mature vinegar, only 556 bacterial OTUs and 172 fungal OTUs were observed [30], while in Sichuan bran vinegar, only found 63 bacterial genera and 41 fungal genera [31]. These results indicated that the SSV *Cupei* had highly variable bacterial and fungi species compositions.

**Fig. 5:**
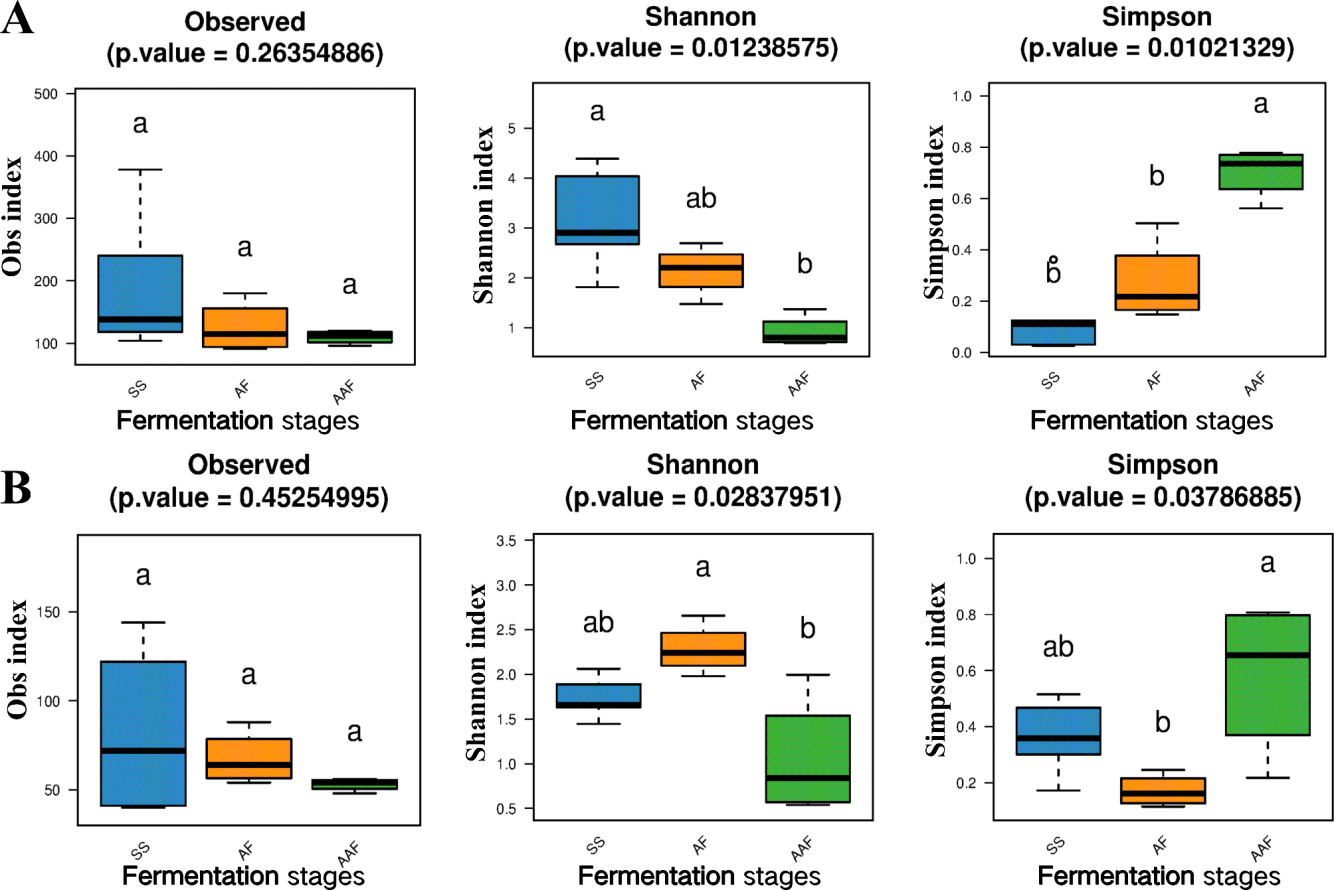
Alpha diversity of microorganism community in *Cupei* samples. Observed species numbers, Shannon and Simpson diversity index for bacterial (**A**) and fungi (**B**). Lowercase a and b marks the difference comparison results between two groups. The same letter indicates that there is no significant difference between the two groups. Different letters represent significant differences between the two groups (*P* < 0.05)

The alpha diversity metrics, including Observed, Shannon and Simpson, were generated to evaluate richness and diversity of microbial community. As shown in Table 2; Fig. 5, the observed index indicated that the water content in the raw material (SS0) sample was lower as fermentation liquid (*Daqu*) had not yet been added, which was more favorable for the growth of fungi, resulting in lower bacterial abundance and higher fungal abundance. Upon addition of *Daqu* (SS1), microorganisms began to proliferate rapidly in the early stages of fermentation, and the abundance and diversity of bacteria continued to increase, reaching a peak on day 7 (SS7), whereas the abundance of fungi was highest on day 1 (SS1), possibly due to the high yeast content in *Daqu*, after which its abundance started to decline. These results suggested that the growth of fungi in the raw materials might be inhibited due to environmental changes during fermentation. As the fermentation time increased, the acetic acid content also increased, which inhibited the growth of acid-intolerant microorganisms and affecting the diversity of microbial communities [19]. As a result, the abundance and diversity of bacteria showed a decreasing trend from day 10 to day 21 of fermentation (SS10-AAF1). Although the acetic acid content continued to increase from day 24 to day 30 of fermentation (AAF4-AAF7), the diversity of bacteria and fungi changed little, indicating that the microbial community had gradually stabilized in the high acid environment during the later stages of fermentation. Throughout the fermentation process, the abundance of the fungal community remained at a low level, which was also reflected in the number of culturable microorganisms (Fig. S2), where the number of fungi was much lower than that of bacteria during SSV fermentation, indicating that bacteria were the dominant microorganisms during SSV fermentation. Additionally, the fluctuation of fungal community diversity may be attributed to the competitive advantage of acid-tolerant bacteria in the growth and reproduction of other microorganisms in strongly acidic environments, or to other fermentation factors such as temperature, nutrients, and pollution [32].

**Table 2:** Alpha diversity of bacterial and fungi community in *Cupei* samples

Samples	16s rDNA (region: V3-V4)			ITS (region: ITS1 ~ ITS2)		
	Observed	Shannon	Simpson	Observed	Shannon	Simpson
SS0	104.00	4.04	0.03	122.00	1.66	0.47
SS1	118.00	1.81	0.36	144.00	1.63	0.52
SS4	138.00	2.68	0.11	40.00	1.45	0.36
SS7	378.00	4.39	0.03	41.00	2.06	0.17
SS10	240.00	2.90	0.12	72.00	1.89	0.30
AF1	132.00	2.25	0.18	69.00	1.98	0.25
AF4	180.00	2.70	0.15	54.00	2.27	0.14
AF7	91.00	2.16	0.25	88.00	2.66	0.12
AF10	97.00	1.48	0.50	59.00	2.21	0.19
AAF1	120.00	1.37	0.56	55.00	2.00	0.22
AAF4	96.00	0.73	0.76	53.00	0.60	0.79
AAF7	107.00	0.88	0.71	48.00	0.54	0.81
AAF10	116.00	0.70	0.78	56.00	1.08	0.52
Mean^SS^	195.60	3.16	0.13	83.80	1.74	0.36
Mean^AF^	125.00	2.14	0.27	67.50	2.28	0.17
Mean^AAF^	109.75	0.92	0.70	53.00	1.05	0.58

^SS^ SS fermentation stage samples; ^AF^ AF fermentation stage samples; ^AAF^ AAF fermentation stage samples

### Changes of microbial community succession during fermentation process

**Fig. 6:**
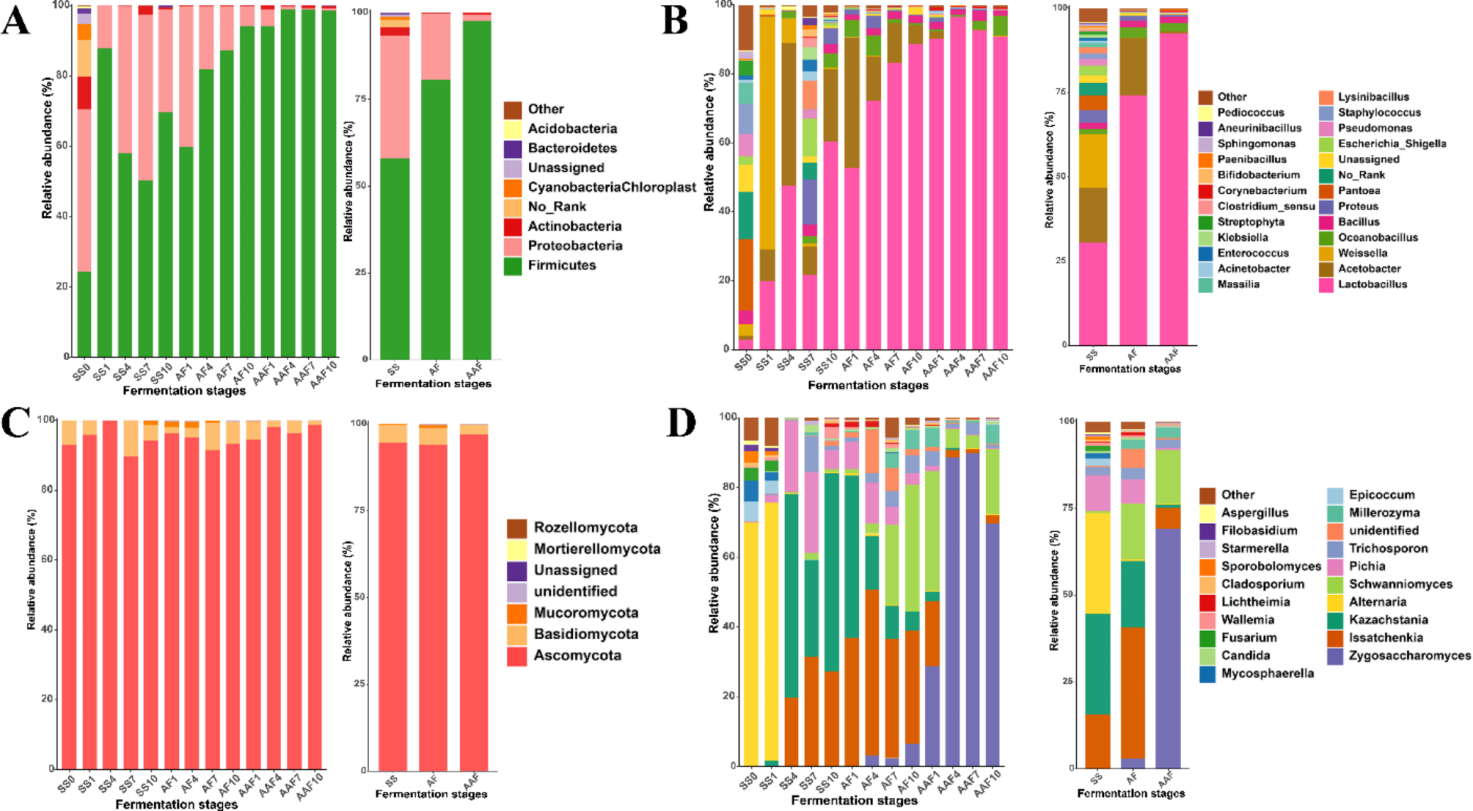
Microbial community compositions in different fermentation stages. The composition of bacteria at phyla (**A**) and genus (**B**) levels and fungi at phyla (**C**) and genus (**D**) levels

Microorganisms played a vital role in the fermentation process of vinegar, which could release various enzymes to accelerate starch saccharification, provide nutrients for the microbial community, and contribute to the flavor of the product [10, 33]. To further identify the structure of microbial communities, the ASVs from 16 S rRNA and ITS sequencing were annotated and classified at phylum and genus levels. For bacteria, *Firmicutes* and *Proteobacteria* were predominant at the phylum level, accounting for more than 97% of total abundance, followed by *Actinobacteria* (1.25%) and *Bacteroidetes* (0.20%) (Fig. 6A). The abundance of *Firmicutes* increased from 24.39 to 98.63%, while the abundance of *Proteobacteria* decreased from 46.02% at the 0d to 0.63% at the 30d. At the genus level of bacteria, an overview of the top 25 genera at three stages was displayed in Fig. 6B. During the three fermentation stages, the predominant populations were *Lactobacillus* (68.02%) and *Acetobacter* (12.58%), followed by *Weissella* (5.84%), *Oceanobacillus* (2.57%), *Pantoea* (2.16%) and *Bacillus* (1.88%). It was noteworthy that the abundances of *Acetobacter* (20.15%) and *Weissella* (17.42%) at the SS stage decreased to 0.55%, 0.04% at the AAF stage, respectively, while *Lactobacillus* (37.55%) increased to 92.50%, becoming the most major genus on AAF stage. In spontaneous fermentation, many factors can affect the growth of *Acetobacter*, which was that some AAB does not tolerate high concentration of acetic acid [34, 35]. Compared with Shanxi aged vinegar [5], the abundance of *Lactobacillus* was only 47.9% at the AAF stage, while 80% of *Lactobacillus* was observed in Tianjin duliu vinegar [8], 49.8% in Zhejiang rose vinegar [36], among of which were lower than SSV. The high abundance of LAB in SSV *Cupei* was also observed in the results of enumeration of culturable bacteria (Fig. S2). Previous studies demonstrated that *Lactobacillus* could produce large amounts of lactic acid, acetic acid, FAAs, and other flavor compounds during fermentation process [37], which indicated that the high abundance of *Lactobacillus* might be very beneficial for the formation of the rich flavor of SSV. Interestingly, *Pantoea* was not detected in any other samples after 4 days of fermentation, and *Lactobacillus* was the only dominant bacteria in the late fermentation period. It indicated that the acetic acid brewing environment had selective pressure on exogenous microorganisms, thereby affecting the microbial diversity [19].

For fungi, the community was dominated by two phyla during the fermentation process, namely, *Ascomycota* (95.06%) and *Basidiomycota* (4.36%) (Fig. 6C). This phenomenon is consistent with the results of Shanxi aged vinegar, Zhenjiang aromatic vinegar and *Ziziphus jujube* Vinegar [16, 23, 38], indicating that *Ascomycota* and *Basidiomycota* might play a key role in vinegar fermentation. At the genus level, the diversity of dominant fungal populations in each fermentation stage were displayed in Fig. 6D. In the early fermentation stage, two kinds of yeast, *Kazachstania* and *Issatchenkia* were the main fungal genera, while in middle and late fermentation stages, three kinds of yeast, *Zygosaccharomyces*, *Schwanniomyces* and *Issatchenkia* were dominant, which was first noticed in our study as the dominant yeast genera in vinegar fermentation process. Interestingly, *Zygosaccharomyces* was not detected in SS0 and SS1 samples, and neither was the main genus in SS and AF stages, but in AAF1 sample (21d), the abundance of this kind of yeast increased to 28.71%, and reached the highest in AAF7 sample (27d), accounting for 89.70%, becoming the most abundant genus. This kind of yeast was not reported as dominant genus in other cereal vinegars, but was reported to be abundant in fermented foods of Pixian Doubanjiang [39] and Reduced-Salt broad bean paste [40], might be contributed to the release of FAAs by autolysis [41]. In our study, FAAs accumulated continuously during the AAF stage (Fig. 4), suggesting a possible relationship with *Zygosaccharomyces*. We also observed *Zygosaccharomyces*, *Schwanniomyces* and *Issatchenkia* were dominant in AAF stage of SSV, which was different from other cereal vinegars, their main yeast genera were *Saccharomyces* and *Saccharomycopsis* [5, 23, 38]. *Issatchenkia* was considered the main microorganism causing aerobic deterioration of cereals [42]. *Saccharomyces* and *Saccharomycopsis* were reported to product alcohol [43] and release amylase [10], respectively. Although some strains of *Schwanniomyces* have been shown to have potential pathogenicity [44], several studies have also suggested that certain strains of *Schwanniomyces* may play a beneficial role in fermentation processes [45–48]. At present, we only observed the structure of the microbial community during the fermentation process (30 days), and the changes of *Schwanniomyces* during the post-ripening process of SSV were not clear. Meanwhile, the specific role of *Schwanniomyces* strains in SSV has not been thoroughly studied. Therefore, it is necessary to conduct a more detailed investigation to determine the specific roles and potential risks associated with the *Schwanniomyces* strains present in SSV to conduct a comprehensive evaluation of its quality and safety.

In summary, the microbial community of *Cupei* samples underwent significant variations during the different stages of vinegar fermentation. At the early stage of fermentation, the microbial communities in *Cupei* were mainly derived from raw materials and *Daqu*, with high diversity. At this time, microorganisms had not yet multiplied in large numbers, so the abundance of *Acetobacter* and *Lactobacillus* was relatively low. As fermentation time increased, the levels of acetic acid and alcohol increased, resulting in the inhibition of some microorganisms, particularly *Weissella* and *Alternaria*. At this time, yeasts, *Lactobacillus* and *Acetobacter* multiplied continuously and increased in abundance. At the later fermentation stage is characterized by further increased in the levels of acetic acid in the fermentation environment (as depicted in Fig. 3), leading to the death of most microorganisms that were intolerant to the strong acid environment. Consequently, the growth of acid-resistant microorganisms was favored, leading to a decrease in the diversity of the microbial community, and *Lactobacillus*, *Zygosaccharomyces*, *Schwanniomyces* were dominant genera in the late fermentation stage.

### Beta diversity analysis and representative microbes on different fermentation stages

**Fig. 7:**
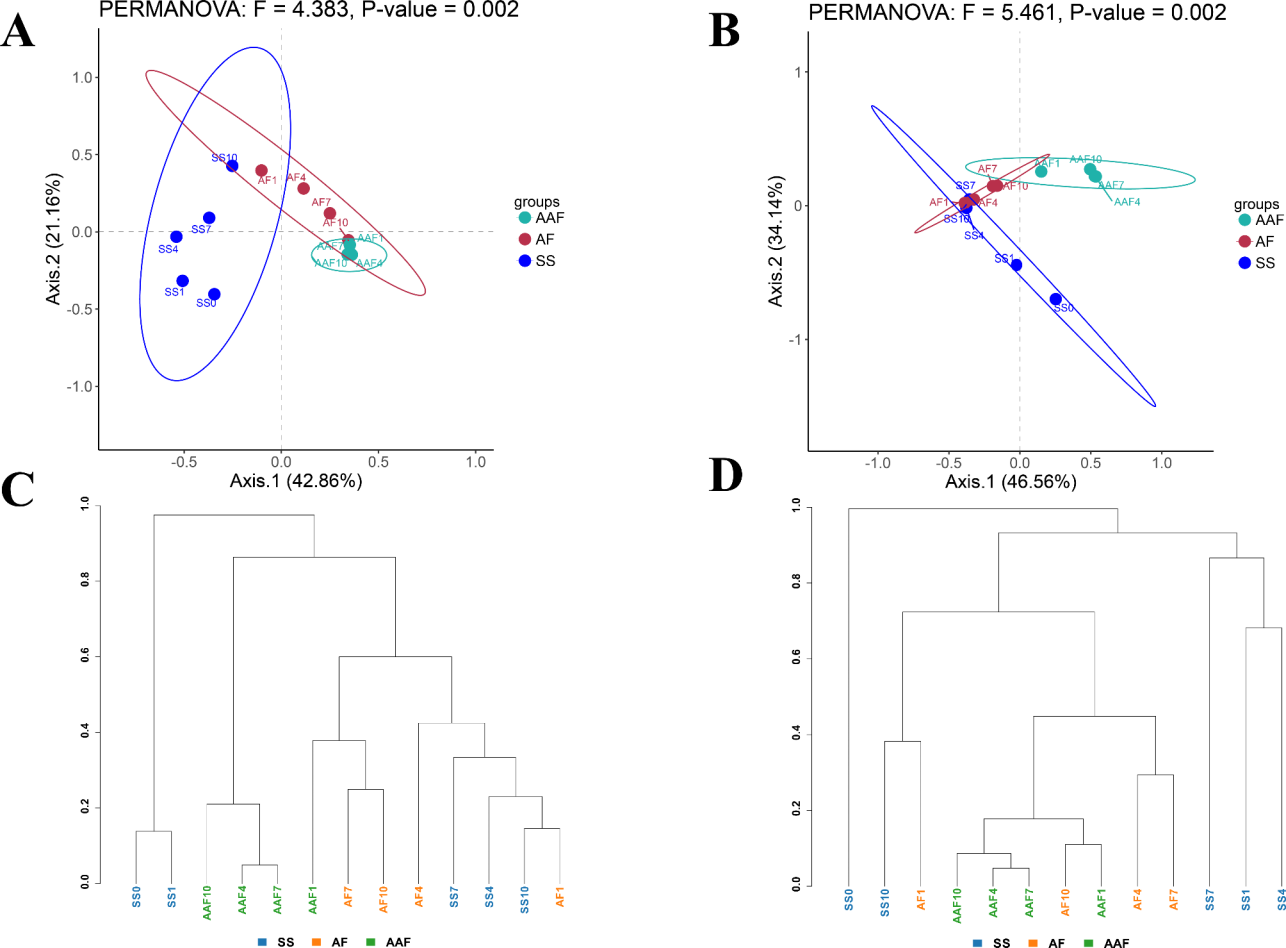
PCoA and UPGMA clustering tree analysis of fermentation samples. PCoA for bacterial (**A**) and fungal (**B**) community; UPGMA clustering tree for bacterial (**C**) and fungal (**D**) community

Although, based on experience, the fermentation process of SSV is divided into starch saccharification, alcohol fermentation and acetic acid fermentation stages for 10 days for each, whether the variation of bacterial and fungal communities supports this classification need to be addressed. Here, the PCoA analysis based on Bray-Curtis distance was used to understand the clustering of SSV *Cupei* samples. As shown in Fig. 7A and B, the first two components explained 64.02% and 80.70% of bacterial and fungal variability, respectively. The bacterial and fungal communities of *Cupei* samples were well separated, indicating that there was a remarkable difference in bacterial and fungal communities in different stages (*P* < 0.01). Compared to samples from SS stage, samples in AF and AAF stages were closer to each other, indicating their similarity in bacterial and fungal composition.

Furthermore, hierarchical clustering analysis was performed using the β diversity distance matrix and the tree branch structure was constructed using unweighted pair-group method with arithmetic means (UPGMA) to further compare the similarities and differences of bacterial and fungal population composition among *Cupei* samples. As illustrated in Fig. 7C and D, the bacterial and fungal communities of the raw material (SS0) samples exhibited significant differences from those of *Cupei* samples at other fermentation stages and were placed in separate branches. In contrast, the samples from the AAF fermentation stage showed close distances, particularly the three samples (AAF4, AAF7, AAF10) in the late fermentation stage that were grouped together in the same branch, indicating that the bacterial and fungal community structures of these samples were similar. These results corroborated the findings of PCoA and provided further insights into the microbial dynamics during the fermentation process. Based on the results of hierarchical clustering analysis of microbial communities, Zhang G et al. divided the solid-state fermentation process of Taiyuanjing sun-dried vinegar into three stages: the first stage lasted from 1 to 8 days, the second stage lasted from 9 to 12 days, and the third stage lasted from 14 to 16 days [49]. Additionally, Rong K et al. have also employed PCoA and UPGMA methods to investigate the differences in microorganisms present in *Cupei* samples between winter and summer in Shanxi old vinegar. The results showed that the microorganisms in the same season were similar and clustered together [30]. In summary, the results of microbial community composition of SSV support, to some extent, an empirically based classification of fermentation stages.

**Fig. 8:**
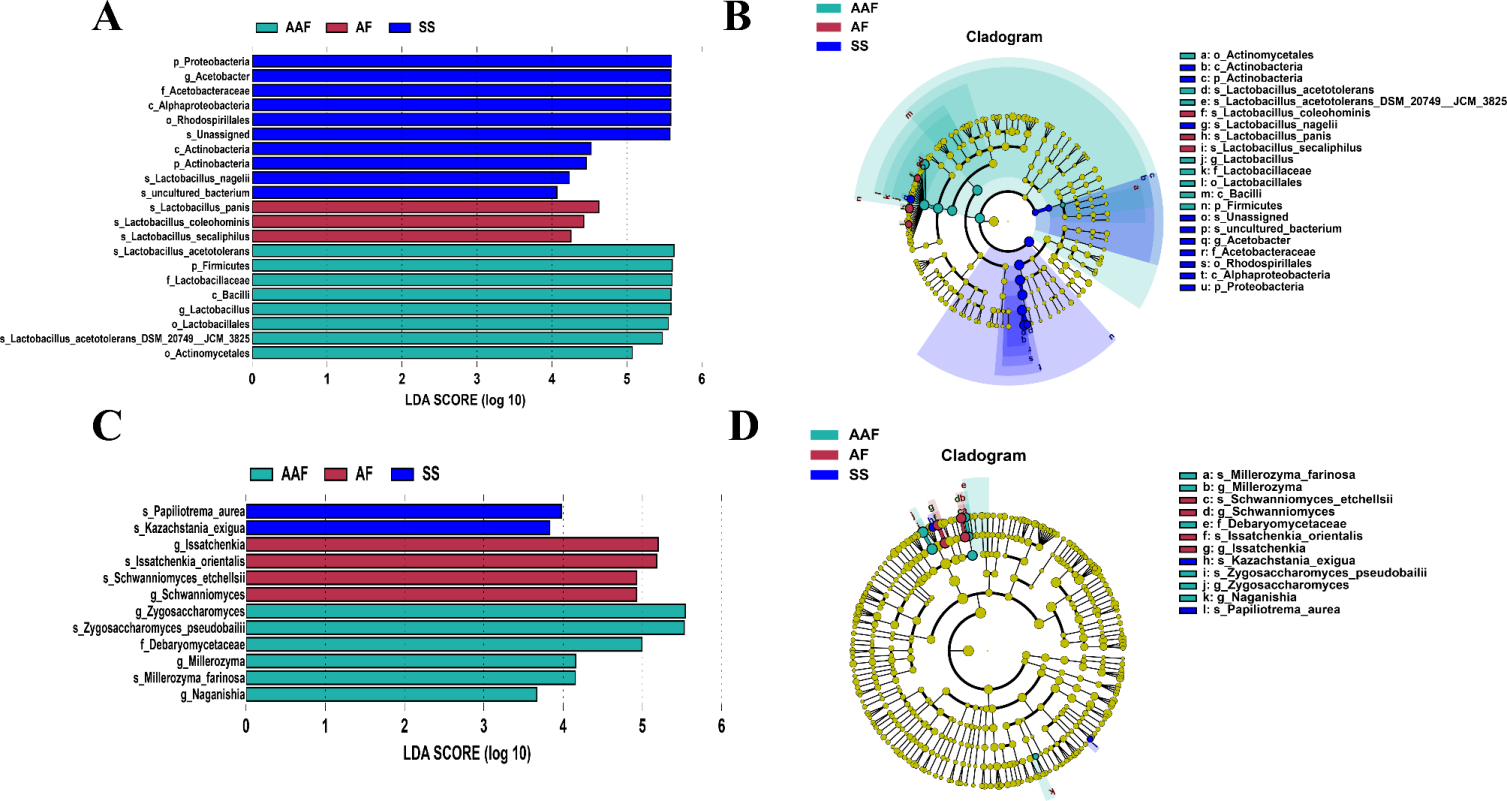
LEfSe analysis on different fermentation stages. LEfSe analysis for bacterial (**A**) and fungal (**C**) community. LEfSe showed a list of specific bacteria and fungi that enable discriminate different fermentation stages. *P* < 0.05 and a default LDA score ≥ 2.0 were considered significant in the Kruskal–Wallis evaluation. Cladogram demonstrating the bacterial (**B**) and fungal (**D**) community with significant differences at different fermentation stages. Blue, red and green represent different stages, with the microbial classifications at the phylum, class, order, family, and genus levels illustrated from inside to outside. The Blue, red and green nodes in the phylogenetic tree represent microorganisms that play important roles in the SS, AF and AAF, respectively. Yellow nodes represent bacteria and fungi with no significant difference

Furthermore, the specific bacterial and fungal taxa within each fermentation stage were identified by the LEfSe analysis, and the threshold on the LDA score for discriminative features was adopted to select the greatest differences in taxa at different stage. The overall comparison of these representatives bacterial and fungi taxa from each time-point during three fermentation stages were conducted by LDA and Cladogram (Fig. 8). In the comparison of the bacterial populations among three stages, 4 bacterial genus and species (*Acetobacter*, *Unassigned*, *Lactobacillus nagelii* and *uncultured bacterium*) from SS, 3 (*Lactobacillus panis*, *Lactobacillus coleohominis* and *Lactobacillus secaliphilus*) from AF and 3 (*Lactobacillus acetotolerans*, *Lactobacillus* and *Lactobacillus acetotolerans_DSM*) from AAF exhibited differentially abundant in each stage (Fig. 8A and B). In the SS stage, *Acetobacter* had the highest LDA score of 5.58, followed by *Unassigned* (5.57), *Lactobacillus nagelii* (4.23). *Lactobacillus panis*, *Lactobacillus coleohominis* and *Lactobacillus secaliphilus* were the significantly enriched species in the AF stage, while the enriched species were only *Lactobacillus acetotolerans* and *Lactobacillus* in the AAF stage. The *Lactobacillus acetotolerans* existed during the entire fermentation process in SSV, and the relative abundance reached 83.4% at the AAF stage, which was very different in Shanxi vinegar and Tianjin vinegar [5, 8]. For fungal LDA analysis, 2, 2 and 2 fungal species were found to be enriched at the SS, AF and AAF stages, respectively. *Papiliotrema aurea* and *Kazachstania exigua* at the SS stage, *Issatchenkia orientalis* and *Schwanniomyces etchellsii* at the AF stage, and *Zygosaccharomyces pseudobailii* and *Millerozyma farinosa* at the AAF stage, were verified to the specific enriched on each stage (Fig. 8C and D). These results were consistent with those in the phylogenetic tree (Fig. S3).

### Correlation between dominant microbes and metabolites during fermentation process

**Fig. 9:**
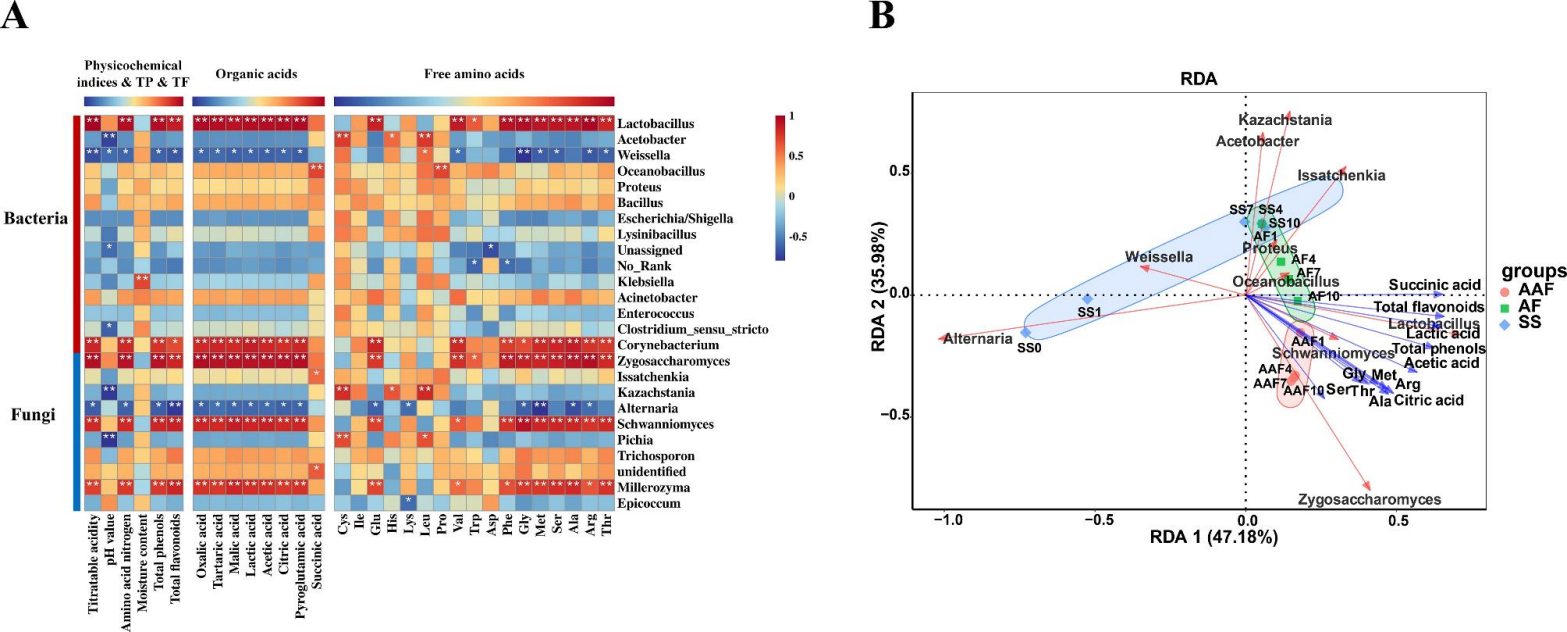
Analysis of correlation between dominant microbes and metabolites. Heatmap showing the correlation between physicochemical indices, total phenols, total flavonoids, organic acids, free amino acids and representative microbial genus (The top 15 bacteria in abundance and the top 10 fungal genera were included.) on different fermentation stages. *P < 0.05; **P < 0.01 (**A**). Redundancy Analysis (RDA) of dominant microorganisms, fermentation time, and major metabolites in *Cupei* samples. The red arrows represent dominant microorganisms, and the blue arrows represent different metabolites. Diamonds, squares and circles represent samples from SS, AF and AAF fermentation stages, respectively (**B**)

To address the relationships between the microbial community succession and the metabolites changes at different fermentation stages, Spearman’s correlation analysis was conducted between top 25 microbial genera and 31 metabolites. As shown in Fig. 9A, positive correlations were observed between 13 genera and metabolites of SSV, while 9 genera represented negative. Among the 13 positive correlations, *Lactobacillus* and *Zygosaccharomyces* showed strongly associated with most of the metabolites (21/31), followed by *Corynebacterium* (20/31), *Schwanniomyces* (20/31) and *Millerozyma* (20/31), indicating that these microorganisms possess a high tolerance for acidic environment. It was reported that *Lactobacillus* could promote the production of malic acid and citric acid through the tricarboxylic acid cycle pathway [50], as well as hydrolyze peptides with specific aminopeptidases to form a variety of amino acids [51], which might contribute to form soft taste and rich nutrition of SSV. Interestingly, although *Corynebacterium* and *Millerozyma* with less abundance, they were still had strong positive correlation with acetic acid, lactic acid, citric acid, etc., which might play an indispensable role in the formation of special flavor. In previous study, the rare microbial taxa have important effects on local microbial interactions in response to environmental disturbances [9, 52].

Additionally, redundancy analysis (RDA) was also conducted to further understand the relationships between dominant microorganisms and major metabolites. In Fig. 9B, axes 1 and axes 2 could explain 83.16% of the data variance of the correlation between microorganisms and metabolites. Furthermore, most of the OAs and FAAs were accumulated at the AAF stage, and showed a highly positive correlation with *Lactobacillus*, *Zygosaccharomyces* and *Schwanniomyces*. These were coincident with the previously described results (Figs. 3 and 4).

It is well known that the interaction among community members and that between the members and the metabolites are extremely complicated. For example, Huang et al. found that the flavor compounds in Zhenjiang aromatic vinegar was related to the core microbiota, including *Acetobacter*, *Lactobacillus*, *Lactococcus*, *Gluconacetobacer*, *Wickerhamomyces* and *Saccharomyces* [38, 53]. And Ai et al. reported that *Acetobacter*, *Lactobacillus*, *Candida*, and *Monascus* were core microbes for the production of volatiles and OAs in Sichuan bran vinegar [31]. Nevertheless, in our study, except for *Lactobacillus*, the core microbes during SSV fermentation were different from other vinegars, such as *Corynebacterium*, *Zygosaccharomyces*, *Schwanniomyces* and *Millerozyma*, which might lead to the difference in the flavor of SSV from other vinegars. This finding will be very helpful for the further study of the relationship between microbial community and unique flavor and taste of SSV.

## Conclusions

In this study, we explored the relationship between metabolites changes and microbial succession along with the special solid-state fermentation craft of SSV by using HPLC and Illumina high-throughput sequencing technology. We found that dominant microorganisms in SSV fermentation process were different from some other traditional Chinese vinegars, especially *Lactobacillus* (relative abundance > 90%) instead of *Acetobacters*, becoming the only dominant genera in the AAF stage, which was also confirmed by the results of culturable methods. Besides, two kinds of yeast, *Zygosaccharomyces* and *Schwanniomyces* were first noticed in our study as the dominant yeast genera. Furthermore, most of OAs, FAAs and other metabolites accumulated at AAF stage. And most of these metabolites demonstrated a highly positive correlation with *Lactobacillus*, *Zygosaccharomyces* and *Schwanniomyces* during the fermentation process. This study is the first report to explore the relationships between microbial diversity and OAs, FAAs and other flavor compounds. Nevertheless, further studies should be performed to explore otherwise important microbes and metabolites, as well as the function of polyphenols, flavonoids and the dominant yeasts of SSV.

## Materials and methods

### SSV fermentation and sample collection

The traditional fermentation process of SSV was performed at Zigong Qiantian Baiwei Food Co., Ltd (Fushun county, Zigong city, Sichuan). Briefly, wheat bran (12,000 kg) was mixed with fresh fermentation starter (*Daqu*) at a ratio of 1:1. The mixture was also called *Cupei*, which was stacked together in the workshop and fermented at room temperature for 30 days with manually mixed every morning to maintain sufficient oxygen and improve acetic acid yield. Besides, the brown leaf mats were applied to cover the *Cupei* to retain the moisture. The fermentation process was divided into three stages: SS, AF, and AAF stages, each lasting for 10 days, which were conducted simultaneously in the same place and followed by transport to the pithos for aging. Samples were collected respectively at SS stage (0d, 1d, 4d, 7d, 10d, marked as SS0, SS1, SS4, SS7, SS10), AF stage (11d, 14d, 17d, 20d, marked as AF1, AF4, AF7, AF10) and AAF stage (21d, 24d, 27d, 30d, marked as AAF1, AAF4, AAF7, AAF10) after stirring at a depth of approximately 30 cm from the surface of *Cupei*. All the samples of approximately 200 g each were collected in triplicate and stored at − 80℃ for the subsequent study.

### Physicochemical features analysis

The moisture content of *Cupei* was determined by direct drying method at 105℃ [39]. In brief, the *Cupei* samples (Approximately 30 g) were homogenized with 90 mL of distilled water, and then the pH value was determined with a pH meter (Fangzhou technology, Chengdu, China). The titratable acidity and amino acid nitrogen were analyzed by titration method using pH as an indicator according to the recommendation of national standard of China GB/T 5009.235 [54]. Briefly, titratable acidity was titrated with 0.01 M NaOH until the final pH of the solution was 8.2. Then, 10 mL formaldehyde was added to the sample to fix the amino group, followed by titration with 0.01 M NaOH until the final pH of the solution reached 9.2. The amount of titrant was calculated to determine the content of titratable acidity and amino acid nitrogen, respectively.

### Organic acid and free amino acids determination

A HPLC system (Thermo Fisher Scientific, Massachusetts, USA) equipped with an Ultimate AQ-C18 (250 mm x 4.6 mm, 5 μm) column was used for identification and analysis of OAs and FAAs. In this study, 8 OAs standards and a mixture of 17 standards FAAs were used for identification and quantification of OAs and FAAs in *Cupei* samples. For OAs determination, *Cupei* samples were pretreated with zinc sulfate and potassium ferricyanide solution, and then the samples were filtered with 0.22 μm microporous membrane. The mobile phase consisted of 20 mmol/L sodium dihydrogen phosphate (NaH_2_PO_4_, pH 2.7), and the flow rate was maintained at 0.8 mL/min. The injection volume was 10 µL, and the detected wavelength of the UV detector was 210 nm. For FAAs determination, pre-column derivatization with phenyl isothiocyanate (PITC) was established [55]. The mobile phase consisted of 20 mmol/L anhydrous sodium acetate - triethylamine solution (pH 6.2, A) and methanol-acetonitrile-aqueous solution (v/v/v = 2:6:2, B), using a gradient program of 95%~52% (A) in 0 ~ 39 min, 52%~0% (A) in 39 ~ 40 min, 0% (A) in 40 ~ 45 min, 0%~95% (A) in 45 ~ 46 min, 95% (A) in 46 ~ 60 min. The flow rate maintained at 0.8 mL/min. The injection volume was 10 µL, and the detected wavelength of the UV detector was 254 nm.

### Total phenols and total flavonoids determination

Since phenols are also important flavor substances in Chinese traditional vinegars. Total phenols (TP) were determined by Folin-Ciocalteu method with chlorogenic acid as the standard according to a previously reported method [56]. In brief, 500 µL of *Cupei* extract sample was mixed with 2.5 mL of Folin-Ciocalteu reagent and incubated at room temperature for 3 min, then 2 mL of Na_2_CO_3_ solution (7.5%, w/v) was added to the mixture, followed by incubation at room temperature for 90 min in the dark. The absorbance of the mixture was measured at 747 nm. And the results were expressed as gram equivalent of chlorogenic acid per kg of samples (g CAE/kg).

Total flavonoids (TF) was determined using a colorimetric method as follows [57]: 400 µL of test sample was mixed with 200 µL of 5% (w/v) NaNO_2_ solution, followed by incubation for 5 min. Subsequently, 200 µL of 10% (w/v) Al(NO_3_)_3_ solution, prepared in 100 g/L, was added to the mixture and left to stand for 5 min. Finally, 800 µL of NaOH (4%, w/v) were added to the mixture. After 15 min, the absorbance of the mixture was measured at 510 nm. The results were expressed as gram equivalent of rutin per kg of samples (g RTE/kg).

### Enumeration of culturable bacteria and fungi

The total number of culturable bacteria and fungi in *Cupei* was determined using a standard viable cell counting method to examine the total Colony Forming Units (CFUs) [54]. Briefly, 25 g *Cupei* sample and 225 mL sterilized 0.9% NaCl solution were firstly placed in the sterilized conical flask and then it was homogenized on an oscillation incubator (Thermo Fisher Scientific, Massachusetts, USA) with shaking at 150 rpm for 10 min followed with 10-fold series dilution. For the enumeration of total bacterial, lactic acid bacteria and fungi, the diluted samples were spread on plate count agar (PCA), MRS agar and rose bengal agar (Beijing Land Bridge Technology Co., Ltd., China), and incubated at 37℃ for 48 h, at 37℃ in anaerobic chamber for 72 h, at 28℃ in mould incubator for 120 h, respectively. The average counts of bacteria and fungi in *Cupei* samples were calculated as colony forming units (CFU) per gram or expressed as log CFU/g [54].

### DNA extraction

All samples were pretreated before DNA extraction. Approximately 2 g of *Cupei* sample was homogenized and grinded using liquid nitrogen [16]. Subsequently, 100 mg of each sample was used to extract total genomic DNA using Super Plant Genomic DNA Kit (TianGen Biotech, Beijing, China) according to the instruction of manufacturer. The integrity of genomic DNA was detected through agarose gel electrophoresis and the concentration and purity of genomic DNA were detected through the Nanodrop 2000 and Qubit 3.0 Spectrophotometer.

### PCR amplification and Illumina NovaSeq sequencing

The V3-V4 hypervariable regions of the 16S rDNA gene were amplified with the forward primers 341F (5’-CCTACGGGNGGCWGCAG-3’) and the reverse primer 805R (5’-GACTACHVGGGTATCTAATCC-3’). For fungi, the internal transcribed spacer (ITS) regions were amplified with the primer ITS1 (5’-CTTGGTCATTTAGAGGAAGTAA-3’) and ITS2 (5’-GCTGCGTTCTTCATCGATGC-3’) [58]. The PCR components were as follows: 10× Toptaq buffer (1 µL), Toptaq DNA Polymerase (0.2 µL), 2.5 mM dNTPs (0.8µL), 10 µM of each Forward and Reverse primer (0.2 µL), DNA Template (1 µL), and up to 10 µL of ddH_2_O. The total volume of the reaction was 10 µL. Amplification conditions consisted of an initial denaturation step at 94 °C for 2 min, followed by 25 cycles consisting of denaturation at 94 °C for 30 s, annealing at 55 °C for 30 s, and extension at 72 °C for 1 min, with a final extension of 10 min at 72 °C. PCR amplicons were purified with Agencourt AMPure XP PCR Purification Beads (Beckman Coulter, Indianapolis, USA) and quantified using the Invitrogen Qubit3.0 Spectrophotometer Kit (Thermo Fisher Scientific, CA, USA). The PCR products were sequenced using the Illlumina NovaSeq platform with NovaSeq Reagent Kit v3 at Genesky Biotechnology Co., Ltd. (Shanghai, China).

### Bioinformatics analysis

The raw read sequences were processed by using QIIME2 with the cutadapt plugin for trimming the adaptor and primer sequences [59], and the DADA2 plugin for quality control as well as identification of amplicon sequence variants (ASVs) [60]. A pre-trained Naive Bayes classifier in RDP (Ribosomal Database Project) (version 11.5) with a confidence threshold of 0.8 was applied for the taxonomic assignments of the representative ASV sequences followed with removing of the chloroplast, chondriosome and unclassified sequences [61–63].

To estimate the species diversity and richness of *Cupei* samples, the three alpha diversity indices (Observed, Shannon and Simpson) were calculated based on the normalized ASV abundance profile by using R software, and the ASV-level were evaluated by using Bray-Curtis-based Principal Coordinate Analysis (PCoA). Subsequently, to identify the representative bacterial and fungal taxa of each phase of *Cupei* samples, the linear discriminant analysis (LDA) effect size (LEfSe) algorithm was performed [58], and Spearman’s rank correlation was applied to explore the correlations between bacteria and fungi, the physicochemical factors and significantly enriched taxa as well. Finally, the correlation between dominant microbes and major metabolites in a multifactorial analysis-of-variance model with redundancy analysis (RDA). The cosine similarity (cos) between genera and variables was utilized to determine their quantitative relationship, with positive (cos > 0) or negative (cos < 0) values indicating the nature of the relationship, and the projection distance of variables on the genus direction reflecting their quantitative association [31]. It was conducted with Hiplot online analysis platform (https://hiplot-academic.com).

### Statistical analysis

Physicochemical indices were expressed as mean ± standard deviations (SD) and analyzed by Prism 8.0 software. The significant differences among different fermentation processes were determined by one-way analysis of variance (ANOVA), and pairwise comparison between groups was analyzed by Tukey’s method. A value of *P* < 0.05 was considered as statistically significantly.

## Electronic supplementary material

Below is the link to the electronic supplementary material.

Supplementary Material 1

Supplementary Material 2

## Data Availability

All data generated or analysed during this study are included in this published article and its supplementary information files. The datasets generated in this study are deposited in the NCBI repository, accession number: PRJNA994050 (https://www.ncbi.nlm.nih.gov/sra/PRJNA994050).

## Abbreviations

SSV: Sichuan Sun-dried vinegar
SS: Starch Saccharification
AF: Alcohol Fermentation
AAF: Acetic Acid Fermentation
LAB: Lactic Acid Bacteria
AAB: Acetic Acid Bacteria
FAAs: Free Amino Acids
OAs: Organic Acids
HPLC: High-Performance Liquid Chromatography
PITC: Phenyl Isothiocyanate
TP: Total Phenols
TF: Total Flavonoids
CAE: Equivalent of Chlorogenic Acid
RTE: Equivalent of Rutin
PCA: Plate Count Agar
CFU: Colony Forming Units
ASVs: Amplicon Sequence Variants
LDA: Linear Discriminant Analysis
LEfSe: Linear Discriminant Analysis Effect Size
RDA: Redundancy Analysis
SD: Standard Deviations
Met: Methionine, Thr:Threonine
Asp: Aspartic acid
Arg: Arginine
Ala: Alanine
Glu: Glutamic acid
Leu: Leucine
Ser: Serine
Gly: glycine
Phe: Phenylalanine
Trp: Tryptophan
Cys: Cystine
Lys: Lysine
Pro: Proline
Val: Valine
Ile: Isoleucine
His: Histidine
