# Supplementary material for: Exploring the correlation of metabolites changes and microbial succession in solid-state fermentation of Sichuan Sun-dried vinegar
Source: BMC Microbiol. 2023 Jul 24;23:197. doi: 10.1186/s12866-023-02947-1 (PMC10364395; doi:10.1186/s12866-023-02947-1)
Supplement: Supplementary file 1 — Supplementary Material 1 [file 12866_2023_2947_MOESM1_ESM.docx]

Supplemental files

Table S1 Physicochemical characteristic of *Cupei* samples

| Samples | Moisture（%） | Titratable acidity（g/kg） | Amino acid nitrogen（g/kg） | pH | TP（g CAE/kg） | TF（g RTE/kg） |
| --- | --- | --- | --- | --- | --- | --- |
| SS0 | 12.58±0.04 | 2.68±0.18 | 0.06±0.02 | 6.58±0.04 | 0.47±0.06 | 0.45±0.04 |
| SS1 | 66.80±0.01 | 3.60±0.27 | 0.21±0.03 | 4.22±0.17 | 0.51±0.04 | 0.53±0.02 |
| SS4 | 60.75±0.18 | 5.04±0.18 | 0.60±0.09 | 4.16±0.18 | 0.86±0.03 | 0.70±0.04 |
| SS7 | 63.51±0.15 | 5.22±0.18 | 0.68±0.03 | 4.12±0.37 | 0.93±0.03 | 0.77±0.02 |
| SS10 | 63.79±0.03 | 6.84±0.09 | 0.78±0.05 | 4.26±0.25 | 1.01±0.02 | 0.80±0.03 |
| AF1 | 64.77±0.08 | 6.84±0.18 | 0.88±0.11 | 4.52±0.12 | 1.17±0.04 | 0.83±0.03 |
| AF4 | 61.97±0.01 | 7.20±0.81 | 0.87±0.19 | 4.40±0.17 | 1.12±0.07 | 0.75±0.04 |
| AF7 | 63.43±0.06 | 8.16±0.36 | 1.03±0.01 | 4.41±0.15 | 1.22±0.03 | 0.83±0.03 |
| AF10 | 61.67±0.14 | 10.02±0.18 | 1.09±0.04 | 4.45±0.12 | 1.27±0.02 | 0.90±0.03 |
| AAF1 | 61.81±0.13 | 10.62±0.09 | 1.15±0.06 | 4.51±0.15 | 1.33±0.03 | 0.85±0.04 |
| AAF4 | 63.10±0.14 | 10.74±0.27 | 1.25±0.01 | 4.53±0.10 | 1.39±0.03 | 0.86±0.04 |
| AAF7 | 63.41±0.23 | 10.98±0.09 | 1.27±0.03 | 4.60±0.09 | 1.37±0.03 | 0.89±0.03 |
| AAF10 | 62.99±0.08 | 10.50±0.18 | 1.31±0.03 | 4.69±0.08 | 1.41±0.04 | 0.88±0.02 |
| Mean^SS^ | 53.48±20.54 | 4.84±1.23 | 0.406±0.28 | 4.67±0.88 | 0.75±0.24 | 0.67±0.12 |
| Mean^AF^ | 62.96±1.24 | 8.06±1.13 | 0.955±0.12 | 4.45±0.05 | 1.20±0.05 | 0.83±0.05 |
| Mean^AAF^ | 62.83±0.61 | 10.71±0.18 | 1.24±0.06 | 4.58±0.07 | 1.37±0.03 | 0.87±0.02 |
| *P*^a^ | 0.06 | 0.006 | 0.020 | 0.895 | 0.008 | 0.042 |
| *P*^b^ | 0.81 | 0.024 | 0.017 | 0.999 | 0.350 | 0.811 |
| *P*^c^ | 0.07 | ＜0.0001 | 0.0008 | 0.899 | 0.0008 | 0.020 |

^SS^ SS fermentation stage samples; ^AF^ AF fermentation stage samples; ^AAF^ AAF fermentation stage samples; ^a^ SS fermentation stage samples vs AF fermentation stage samples (*P* < 0.05); ^b^ AF fermentation stage samples vs AAF fermentation stage samples (*P* < 0.05); ^c^ AAF fermentation stage samples vs SS fermentation stage samples (*P* < 0.05).

Table S2 Organic acids contents of *Cupei* samples (g/kg)

| **Samples** | **Oxalic acid** | **Tartaric acid** | **Malic acid** | **Lactic acid** | **Acetic acid** | **Citric acid** | **Pyroglutamic acid** | **Succinic acid** | Total |
| --- | --- | --- | --- | --- | --- | --- | --- | --- | --- |
| SS0 | 0.03±0.003 | 0.22±0.03 | 0.28±0.04 | ND | ND | 0.11±0.01 | 0.01±0.001 | 0.89±0.04 | 1.54±0.30 |
| SS1 | 0.04±0.002 | 0.32±0.03 | 0.33±0.02 | 2.52±0.04 | 0.85±0.04 | 0.13±0.03 | 0.02±0.004 | 1.27±0.02 | 5.48±0.80 |
| SS4 | 0.05±0.006 | 0.48±0.03 | 0.49±0.01 | 3.52±0 | 3.01±0.17 | 0.27±0.02 | 0.06±0.004 | 3.18±0.19 | 11.06±1.45 |
| SS7 | 0.05±0.007 | 0.52±0.01 | 0.55±0.02 | 3.85±0.22 | 3.72±0.03 | 0.59±0.04 | 0.12±0.008 | 3.48±0.07 | 12.86±1.62 |
| SS10 | 0.06±0.001 | 0.53±0 | 0.58±0.01 | 4.71±0.34 | 4.86±0.25 | 0.88±0.06 | 0.17±0.011 | 4.45±0.63 | 16.25±2.06 |
| AF1 | 0.07±0.001 | 0.53±0.01 | 0.59±0.05 | 4.66±0.16 | 5.63±0.17 | 0.97±0.01 | 0.2±0.016 | 4.9±0.29 | 17.56±2.25 |
| AF4 | 0.08±0.01 | 0.55±0.01 | 0.64±0.04 | 4.78±0.36 | 5.81±0.23 | 0.92±0.06 | 0.21±0.019 | 4.73±0.21 | 17.72±2.27 |
| AF7 | 0.08±0.001 | 0.59±0.01 | 0.7±0.03 | 4.97±0.07 | 7.45±0.19 | 1.55±0.03 | 0.3±0.006 | 4.52±0.35 | 20.15±2.58 |
| AF10 | 0.09±0.005 | 0.58±0.01 | 0.72±0.03 | 5.34±0.04 | 8.41±0.1 | 1.77±0.07 | 0.34±0.014 | 4.44±0.45 | 21.68±2.83 |
| AAF1 | 0.1±0.001 | 0.62±0.03 | 0.72±0.07 | 5.74±0.47 | 9.05±0.01 | 2.16±0.01 | 0.41±0.001 | 4.35±0.09 | 23.16±3.01 |
| AAF4 | 0.11±0.002 | 0.72±0.06 | 0.72±0.05 | 5.54±0.33 | 8.91±0.13 | 2.17±0.19 | 0.41±0.01 | 4.49±0.24 | 23.06±2.95 |
| AAF7 | 0.11±0.003 | 0.8±0.04 | 0.75±0.06 | 5.79±0.3 | 10.25±0.78 | 2.32±0.14 | 0.43±0.036 | 4.63±0.32 | 25.07±3.32 |
| AAF10 | 0.12±0.004 | 0.88±0.07 | 0.77±0.02 | 5.88±0.51 | 10.86±0.62 | 2.43±0.15 | 0.43±0.047 | 4.5±0.03 | 25.87±3.47 |
| Mean^SS^ | 0.04±0.01^a^ | 0.42±0.12 | 0.45±0.19 ^a^ | 2.92±0.16 ^a^ | 2.45±1.81^a^ | 0.40±0.30^a^ | 0.08±0.06^a^ | 2.65±1.36^a^ | 9.44±5.27^a^ |
| Mean^AF^ | 0.08±0.01^b^ | 0.56±0.02^b^ | 0.66±0.05 | 4.94±0.26 | 6.83±1.12^b^ | 1.30±0.36^b^ | 0.26±0.06^b^ | 4.65±0.18 | 19.28±1.73^b^ |
| Mean^AAF^ | 0.11±0.01^c^ | 0.76±0.10^c^ | 0.74±0.02 ^c^ | 5.74±0.13 ^c^ | 9.77±0.82^c^ | 2.27±0.11^c^ | 0.42±0.01^c^ | 4.49±0.10^c^ | 24.29±1.21^c^ |

ND：Not Detect; ^SS^ SS fermentation stage samples; ^AF^ AF fermentation stage samples; ^AAF^ AAF fermentation stage samples; ^a^ SS fermentation stage samples vs AF fermentation stage samples (*P* < 0.05); ^b^ AF fermentation stage samples vs AAF fermentation stage samples (*P* < 0.05); ^c^ AAF fermentation stage samples vs SS fermentation stage samples (*P* < 0.05).

Table S3 Free amino acids contents of *Cupei* samples（mg/100g）

| **Samples** | Phe | Ala | Gly | Glu | Cys | Met | Arg | Lys | Leu |
| --- | --- | --- | --- | --- | --- | --- | --- | --- | --- |
| SS0 | ND | ND | ND | ND | ND | 4.51±0.28 | ND | ND | ND |
| SS1 | 0.71±0.08 | 2.02±0.31 | 1.24±0.18 | 0.12±0.06 | 0.99±0.11 | 3.71±0.81 | 4.10±0.70 | 0.25±0.03 | 1.41±0.03 |
| SS4 | 0.66±0.09 | 8.98±0.92 | 2.05±0.28 | 0.06±0.02 | 3.7±0.45 | 22.97±0.32 | 13.57±2.61 | 1.29±0.09 | 2.24±0.07 |
| SS7 | 0.48±0.13 | 39.1±2.79 | 3.01±0.27 | 0.20±0.14 | 2.86±0.41 | 29.95±3.53 | 9.37±1.16 | 0.59±0.18 | 1.97±0.05 |
| SS10 | 6.93±0.43 | 25.06±1.92 | 5.49±0.71 | 0.35±0.18 | 4.32±0.48 | 36.63±3.01 | 15.15±0.69 | 0.78±0.25 | 2.77±0.22 |
| AF1 | 11.53±1.98 | 22.57±1.43 | 6.17±0.49 | 0.08±0.06 | 1.28±0.15 | 14.21±1.48 | 80.91±4.10 | 0.39±0.07 | 2.12±0.21 |
| AF4 | 12.54±0.27 | 28.52±2.30 | 12.39±1.36 | 0.07±0.05 | 1.42±0.18 | 15.34±0.43 | 77.38±2.55 | 0.42±0.12 | 1.73±0.13 |
| AF7 | 19.78±0.87 | 69.57±2.16 | 62.2±1.98 | 0.63±0.04 | 0.11±0.06 | 54.74±2.35 | 120.52±3.61 | 1.03±0.07 | 0.72±0.04 |
| AF10 | 15.62±2.93 | 66.63±5.40 | 67.18±11.01 | 0.44±0.05 | 0.49±0.28 | 72.66±15.39 | 115.76±19.99 | 1.08±0.17 | 0.87±0.11 |
| AAF1 | 17.71±3.96 | 73.89±5.37 | 69.25±11.06 | 0.51±0.20 | 0.71±0.23 | 88.80±1.63 | 121.26±18.05 | 0.34±0.12 | 0.99±0.16 |
| AAF4 | 24.03±1.95 | 88.78±2.76 | 84.28±2.53 | 0.66±0.18 | 0.99±0.66 | 113.56±7.05 | 148.65±4.76 | 0.96±0.40 | 0.83±0.06 |
| AAF7 | 26.82±0.96 | 93.52±3.63 | 42.71±5.98 | 0.87±0.22 | 0.65±0.45 | 56.23±12.30 | 142.15±5.93 | 0.68±0.12 | 1.34±0.56 |
| AAF10 | 28.11±2.43 | 109.14±3.28 | 34.32±6.04 | 0.71±0.08 | 0.31±0.17 | 77.40±12.57 | 140.89±9.70 | 0.71±0.79 | 0.86±0.16 |
| Mean^SS^ | 1.76±2.60^a^ | 15.03±14.91 | 2.36±1.85^a^ | 0.15±0.12 | 2.37±1.63 | 19.55±13.33 | 8.44±5.70^a^ | 0.58±0.44 | 1.68±0.95 |
| Mean^AF^ | 14.87±3.21^b^ | 46.82±21.41^b^ | 36.99±27.85 | 0.31±0.24 | 0.83±0.54 | 39.24±25.27 | 98.64±19.61^b^ | 0.73±0.33 | 1.36±0.58 |
| Mean^AAF^ | 24.17±4.01^c^ | 91.33±12.58^c^ | 57.64±20.07^c^ | 0.69±0.13^c^ | 0.67±0.24 | 84.00±20.69^c^ | 138.24±10.23^c^ | 0.67±0.22 | 1.01±0.20 |

**Continue to Table S3**

| **Samples** | Pro | Trp | Ser | Thr | Asp | Val | Ile | His | Total |
| --- | --- | --- | --- | --- | --- | --- | --- | --- | --- |
| SS0 | ND | ND | ND | 15.20±0.05 | 9.93±0.20 | ND | ND | ND | 29.64±4.19 |
| SS1 | 0.45±0.02 | 0.42±0.10 | ND | 5.43±0.93 | 2.94±0.77 | ND | ND | 0.85±0.13 | 24.64±1.60 |
| SS4 | 0.58±0.03 | 0.51±0.02 | ND | 20.23±2.21 | 6.29±0.99 | ND | 1.03±0.08 | 2.77±0.58 | 86.93±6.99 |
| SS7 | 0.50±0.03 | 0.12±0.14 | 0.53±0.06 | 25.67±3.93 | 6.21±0.54 | 0.12±0.01 | 0.77±0.06 | 1.86±0.06 | 123.31±11.74 |
| SS10 | 0.82±0.07 | 0.56±0.24 | 2.27±0.54 | 28.35±2.78 | 7.67±1.01 | 0.83±0.15 | 1.56±0.21 | 2.05±0.18 | 141.59±10.85 |
| AF1 | 1.45±0.39 | 1.24±0.18 | 2.87±0.66 | 93.07±7.84 | 11.90±1.98 | 0.94±0.28 | 0.27±0.16 | 1.51±0.18 | 252.51±27.12 |
| AF4 | 0.72±0.09 | 0.38±0.15 | 5.16±0.51 | 66.92±2.77 | 8.45±0.99 | 0.59±0.13 | 0.32±0.18 | 0.42±0.03 | 232.77±22.68 |
| AF7 | 0.48±0.82 | 0.92±0.13 | 20.86±0.93 | 126.55±7.03 | 6.90±0.40 | 0.83±0.18 | 0.95±0.57 | 0.80±0.05 | 487.59±41.40 |
| AF10 | 0.90±0.22 | 0.80±0.33 | 15.17±2.81 | 94.64±14.74 | 4.76±1.01 | 0.81±0.50 | 0.45±0.35 | 0.57±0.18 | 458.83±38.12 |
| AAF1 | 0.36±0.08 | 0.49±0.14 | 22.24±5.28 | 97.41±10.82 | 8.66±2.57 | 1.70±0.70 | 0.78±0.46 | 0.72±0.29 | 505.82±40.71 |
| AAF4 | 0.14±0.52 | 0.66±0.55 | 20.41±0.38 | 90.87±1.95 | 7.43±0.51 | 3.55±0.34 | 1.55±0.60 | 1.12±0.91 | 588.47±47.86 |
| AAF7 | 0.99±0.09 | 4.96±0.20 | 88.94±3.58 | 201.76±2.98 | 17.99±0.35 | 2.63±0.24 | 0.72±0.45 | 1.02±0.16 | 683.98±57.45 |
| AAF10 | 0.67±0.39 | 9.74±0.86 | 94.75±6.79 | 182.72±10.01 | 20.96±2.66 | 3.11±0.40 | 0.24±1.58 | 0.71±0.10 | 705.35±56.03 |
| Mean^SS^ | 0.47±0.27 | 0.32±0.22 | 0.56±0.88^a^ | 18.98±8.15^a^ | 6.61±2.27 | 0.19±0.32^a^ | 0.67±0.60 | 1.51±0.97 | 81.22±6.49^a^ |
| Mean^AF^ | 0.89±0.36 | 0.84±0.31 ^b^ | 11.02±7.33^b^ | 95.30±21.14 | 8.00±2.60 | 0.79±0.13^b^ | 0.5±0.27 | 0.83±0.42 | 357.93±31.42^b^ |
| Mean^AAF^ | 0.54±0.32 | 3.96±3.79 ^c^ | 56.59±35.33^c^ | 143.19±49.56^c^ | 13.76±5.83^c^ | 2.75±0.69^c^ | 0.82±0.47 | 0.89±0.18 | 620.91±48.30^c^ |

ND：Not Detect; ^SS^ SS fermentation stage samples; ^AF^ AF fermentation stage samples; ^AAF^ AAF fermentation stage samples; ^a^ SS fermentation stage samples vs AF fermentation stage samples (*P* < 0.05); ^b^ AF fermentation stage samples vs AAF fermentation stage samples (*P* < 0.05); ^c^ AAF fermentation stage samples vs SS fermentation stage samples (*P* < 0.05).
