# Supplementary material for: Exploring the correlation of metabolites changes and microbial succession in solid-state fermentation of Sichuan Sun-dried vinegar
Source: BMC Microbiol. 2023 Jul 24;23:197. doi: 10.1186/s12866-023-02947-1 (PMC10364395; doi:10.1186/s12866-023-02947-1)
Supplement: Supplementary file 2 — Supplementary Material 2 [file 12866_2023_2947_MOESM2_ESM.docx]

Supplemental files


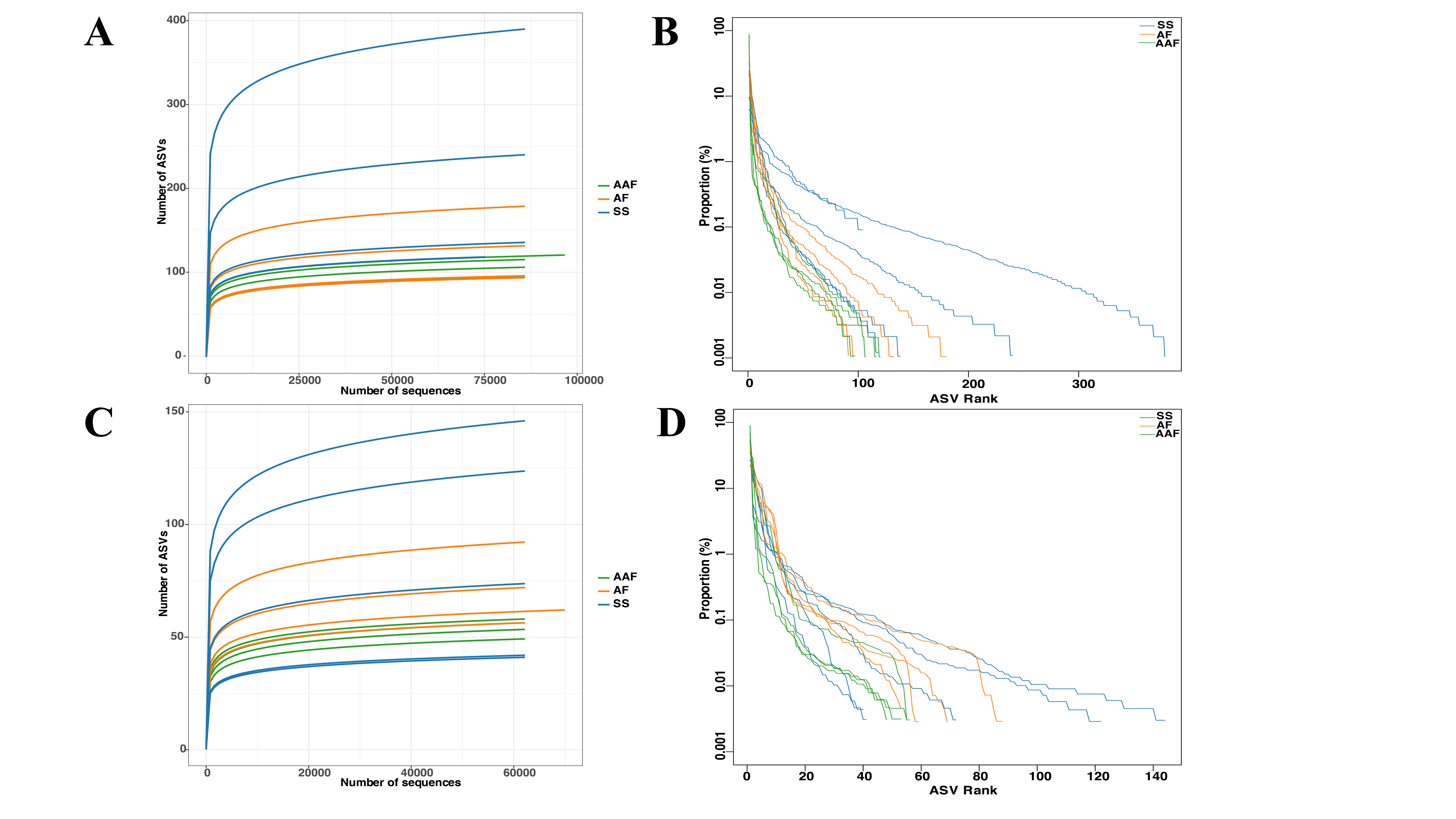


**Fig. S1** Rarefaction and rank-abundance curves for bacteria (A and B) and fungi (C and D).


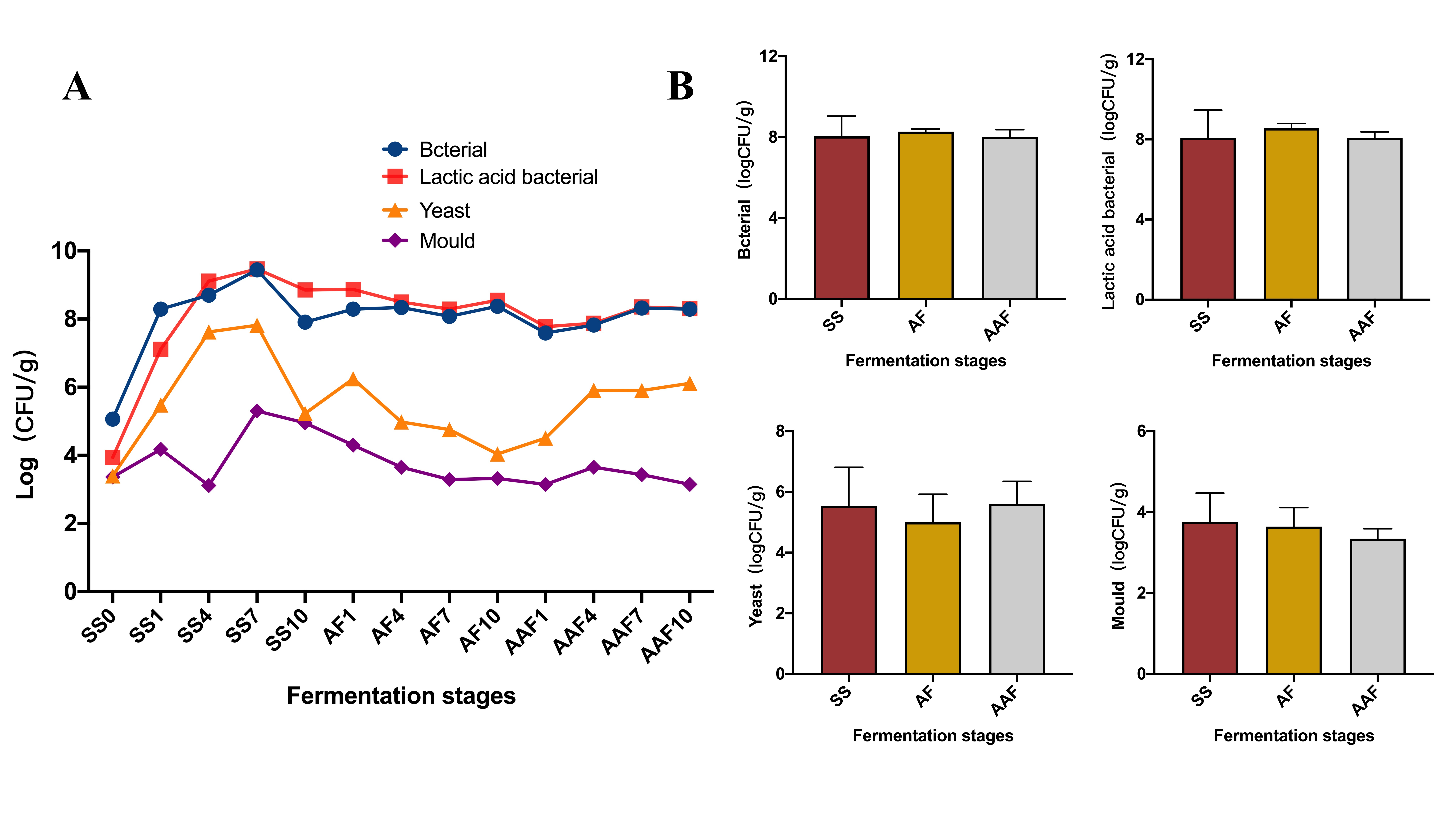


**Fig. S2.** The number of culturable microbial of *Cupei* samples in different fermentation stages. Data were given as mean ± standard deviations.


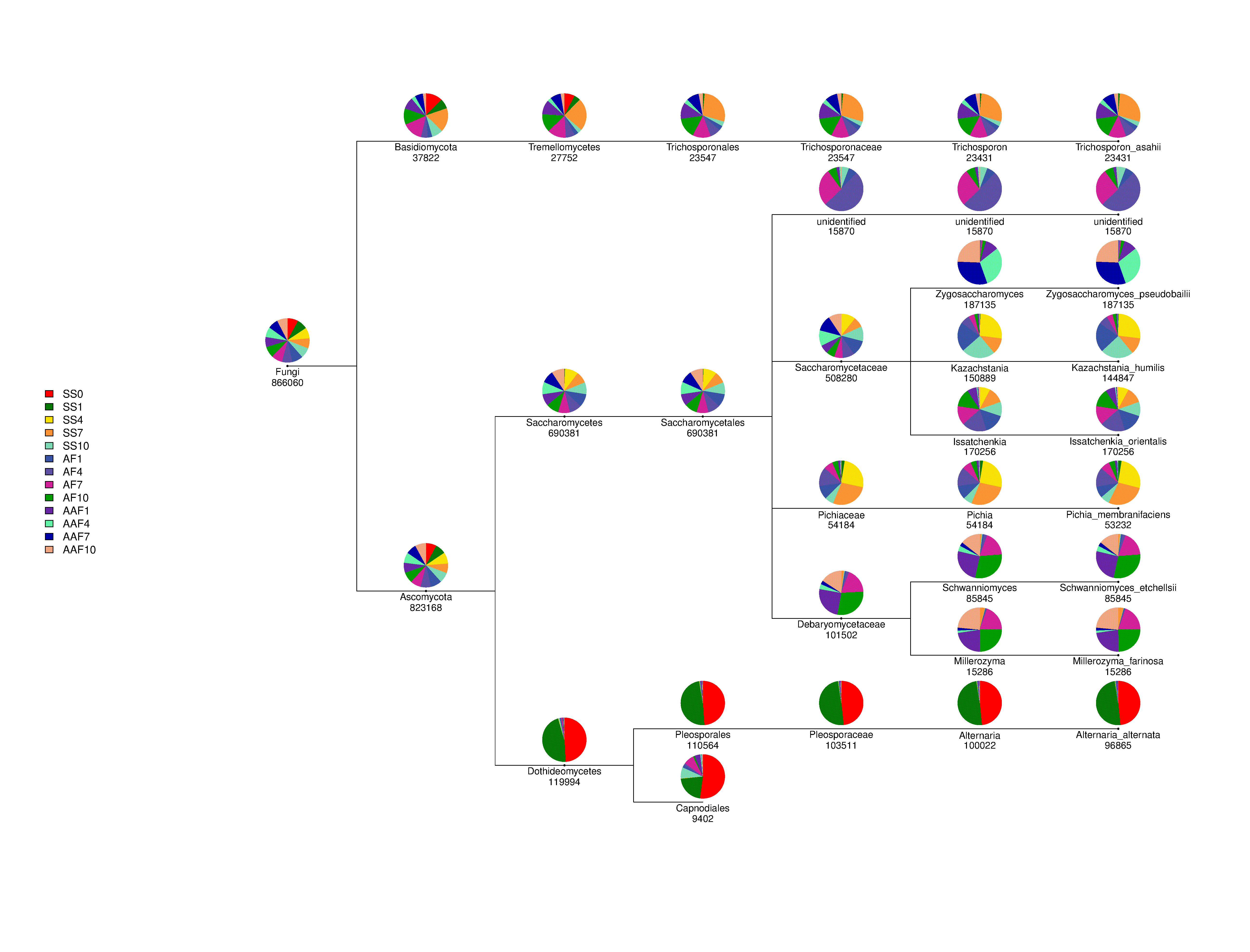

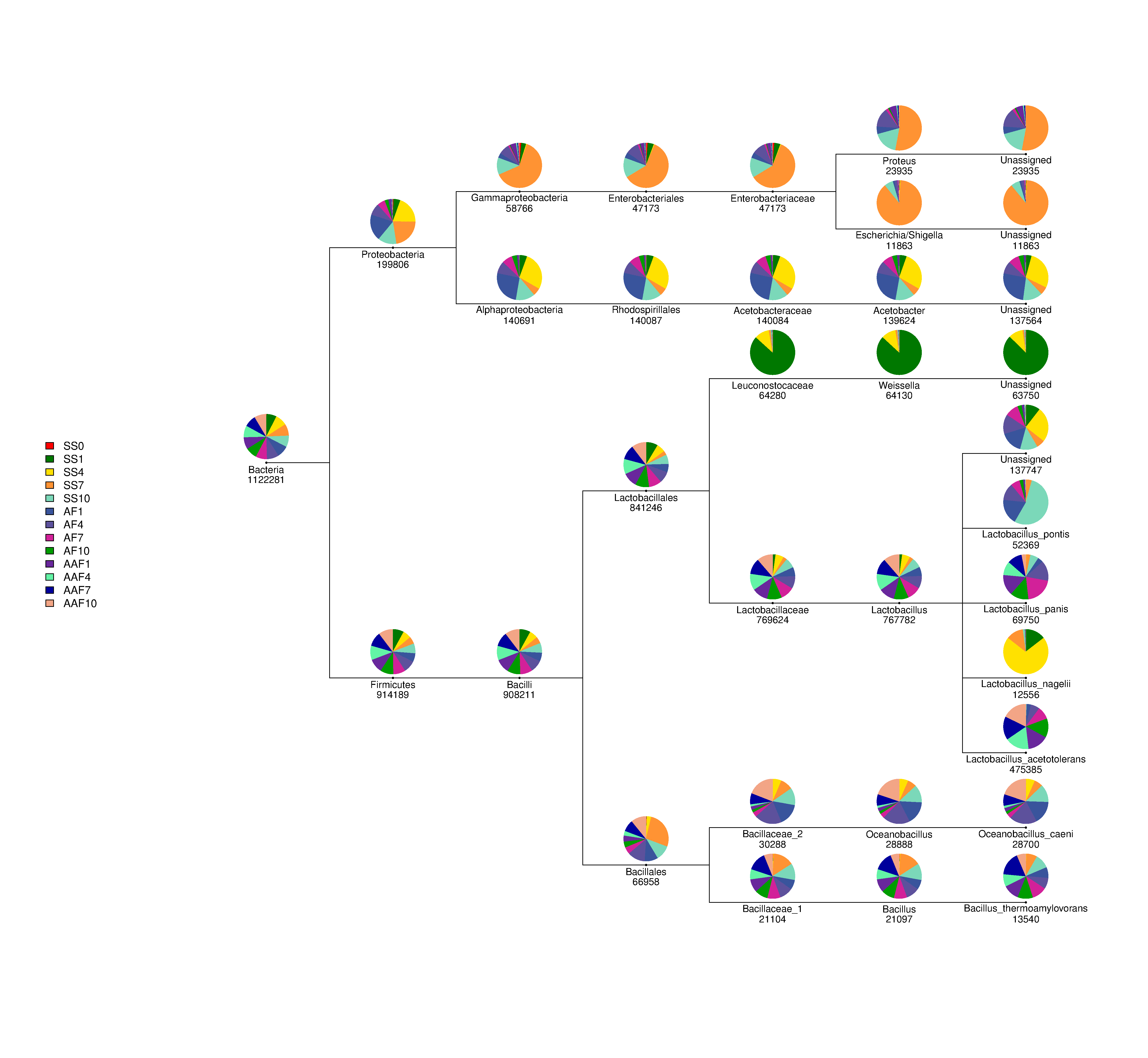
**Fig. S3.** Dendrogram of taxonomic system composition for bacteria (A ) and fungi (B). From the left root node to the right leaf node, each level represents a taxonomic level (from left to right, it is kingdom, phylum, class, order, family, genus, and species level). For each level of species (relative abundance greater than 1%), pie charts were used to show the proportion of that species in each sample. Different colors represent different samples, and a larger fan area of a color means that the sample contains more sequences of that species than the other samples. Numbers under circles indicate the sum of sequences for that species in all samples.

**B**

**A**
